# TMEM106A inhibits enveloped virus release from cell surface

**DOI:** 10.1016/j.isci.2022.103843

**Published:** 2022-02-01

**Authors:** Dexin Mao, Feixiang Yan, Xiaolin Zhang, Guangxia Gao

**Affiliations:** 1 CAS Key Laboratory of Infection and Immunity, Institute of Biophysics, Chinese Academy of Sciences, 15 Datun Road, Chaoyang District, Beijing 100101, China; 2 University of Chinese Academy of Sciences, Beijing 100049, China

**Keywords:** Molecular biology, Virology

## Abstract

Enveloped viruses pose constant threat to hosts from ocean to land. Virion particle release from cell surface is a critical step in the viral life cycle for most enveloped viruses, making it a common antiviral target for the host defense system. Here we report that host factor TMEM106A inhibits the release of enveloped viruses from the cell surface. TMEM106A is a type II transmembrane protein localized on the plasma membrane and can be incorporated into HIV-1 virion particles. Through intermolecular interactions of its C-terminal domains on virion particle and plasma membrane, TMEM106A traps virion particles to the cell surface. HIV-1 Env interacts with TMEM106A to interfere with the intermolecular interactions and partially suppresses its antiviral activity. TMEM106A orthologs from various species displayed potent antiviral activity against multiple enveloped viruses. These results suggest that TMEM106A is an evolutionarily conserved antiviral factor that inhibits the release of enveloped viruses from the cell surface.

## Highlights

• Type II transmembrane protein TMEM106A can be incorporated into virion particles
• TMEM106A inhibits enveloped virion release through C-terminal molecular interactions
• HIV-1 envelope protein interacts with TMEM106A and suppresses its antiviral activity
• TMEM106A is an evolutionarily conserved antiviral factor against multiple viruses

## Graphical abstract

**Figure:**
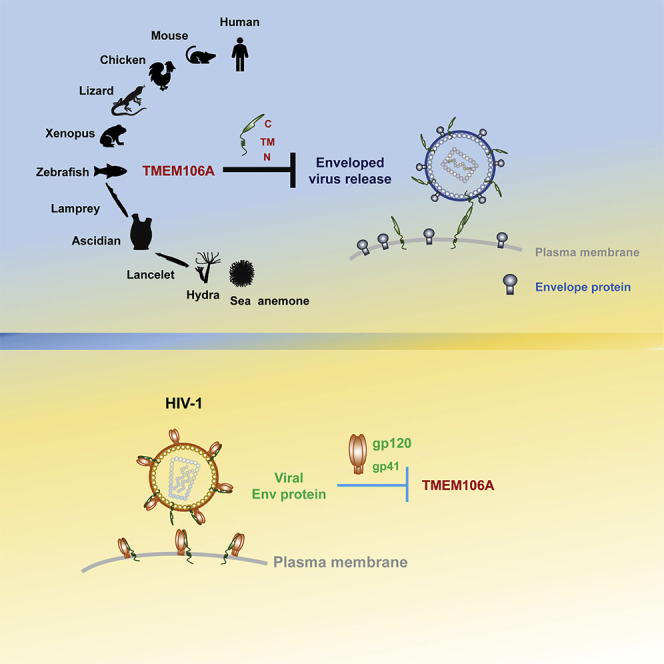

## Introduction

Viruses pose constant threat to hosts from ocean to land. In response to the threat, hosts have evolved a variety of defense mechanisms. The host detects the pathogen-associated molecular patterns of the invading viruses through pattern recognition receptors (PRRs) to initiate antiviral responses (Crowl et al., 2017; Wu and Chen, 2014). In higher vertebrates, activation of the PRRs leads to the induction of type I interferons (IFNs), which subsequently upregulate the expression of hundreds of interferon stimulated genes (ISGs) to inhibit viral replication (Akira et al., 2006; Goubau et al., 2013; Krause and Pestka, 2005; Langevin et al., 2013; Randall and Goodbourn, 2008; Santhakumar et al., 2017; Schoggins and Rice, 2011; Schultz et al., 2004; Tian et al., 2019). The jawless vertebrates and invertebrates do not have the IFN system and use other defense effector mechanisms, such as autophagy (Gui et al., 2019), apoptosis (Mosallanejad et al., 2014) and RNA interference (Wang et al., 2015).

Enveloped viruses are a large group of viruses whose viral cores are surrounded by lipid membrane envelope. The envelope is acquired when the viruses are produced from the host cells. Some enveloped viruses, such as retroviruses and alphaviruses, acquire the envelope from the plasma membrane, whereas others, such as herpesviruses and coronaviruses, acquire the envelope from the intracellular membrane but the virion release process may also involve the plasma membrane. This common feature of enveloped viruses suggests that the virion release process would be a good target for the host defense system to inhibit the replication of enveloped viruses.

BST2 is the most extensively studied host antiviral factor that inhibits virion release of a variety of enveloped viruses (Evans et al., 2010). BST2 is a type II membrane protein containing two membrane anchoring sites and can be incorporated into virion particles (Neil et al., 2008; Perez-Caballero et al., 2009; Van Damme et al., 2008). The two membrane anchoring sites and intermolecular interactions allow the protein to link the virion particle to the cell membrane to inhibit virion release. In addition to BST2, two host factors have been reported to inhibit HIV-1 virion release. The T cell immunoglobulin and mucin domain (TIM) proteins are phosphatidylserine (PS)-binding proteins. Although TIM proteins have been reported to interact with PS on virus surface, promote virion internalization, and thus enhance the infection of a range of enveloped viruses (Jemielity et al., 2013; Meertens et al., 2012; Moller-Tank et al., 2013), they have also been reported to inhibit virion release of HIV-1 and other viruses (Li et al., 2014, 2019). The human mannose receptor C-type 1 (hMRC1) recognizes high-mannose oligosaccharides, which are common patterns on HIV-1 and other viruses, and mediates endocytosis of these pathogens by macrophage (Azad et al., 2014). It has also been reported to retain HIV-1 virion particles on the cell surface (Sukegawa et al., 2018). Bioinformatics analysis revealed that BST2 is conserved only in placental mammals and that TIMs and MRC1 are conserved in higher vertebrates. It is not very clear whether the lower vertebrate and invertebrate hosts also have an antiviral mechanism targeting the virion release process of enveloped viruses.

TMEM106 is a family of transmembrane proteins with three members, TMEM106A, 106B, and 106C. TMEM106A is a type II transmembrane protein primarily localized on the plasma membrane (Xu et al., 2014). It has been reported to function as a tumor suppressor in gastric cancer (Xu et al., 2014), renal cell carcinomas (Wu et al., 2017), and non-small cell lung carcinoma (Liu and Zhu, 2018). TMEM106A inhibits cancer cell proliferation and migration by inducing apoptosis (Liu and Zhu, 2018; Wu et al., 2017; Xu et al., 2014). TMEM106B is also a type II transmembrane protein. But unlike TMEM106A, TMEM106B is primarily localized on the membranes of endosomes or lysosomes; it affects lysosome size, acidification, function, and transport (Nicholson and Rademakers, 2016). Recently, TMEM106B was identified as host factor required for the replication of severe acute respiratory syndrome coronavirus 2 (SARS-CoV-2) (Baggen et al., 2021; Schneider et al., 2021; Wang et al., 2021). The information about TMEM106C is very limited. Bioinformatics analysis reveals that, unlike TMEM106A or TMEM106B, human TMEM106C has two transmembrane domains. TMEM106C may be associated with diseases of joint stiffness and irregular muscle development, such as distal arthropathy and ankylosing spondylitis (Assassi et al., 2011; Genini et al., 2006).

In the present study, we identified human TMEM106A as a host antiviral factor that inhibits the release of multiple enveloped viruses from the cell surface. Human TMEM106A orthologs are found in species as diverse as aquatic invertebrates and mammals. Although in some species they are named TMEM106B or106C in the literature, bioinformatics analysis revealed that they are all type II transmembrane proteins containing one transmembrane domain and an extracellular C-terminal domain. Our fluorescence-activated cell sorting (FACS) analysis confirmed the topology; they are all localized on the cell surface with the C-terminal domain outside, like human TMEM106A rather than TMEM106B or TMEM106C. Functional analysis revealed that overexpression of these orthologs all inhibit the production of MLV, suggesting that TMEM106A is an evolutionarily conserved antiviral factor.

## Results

### TMEM106A is a host antiviral factor that inhibits HIV-1 replication

**Figure 1:**
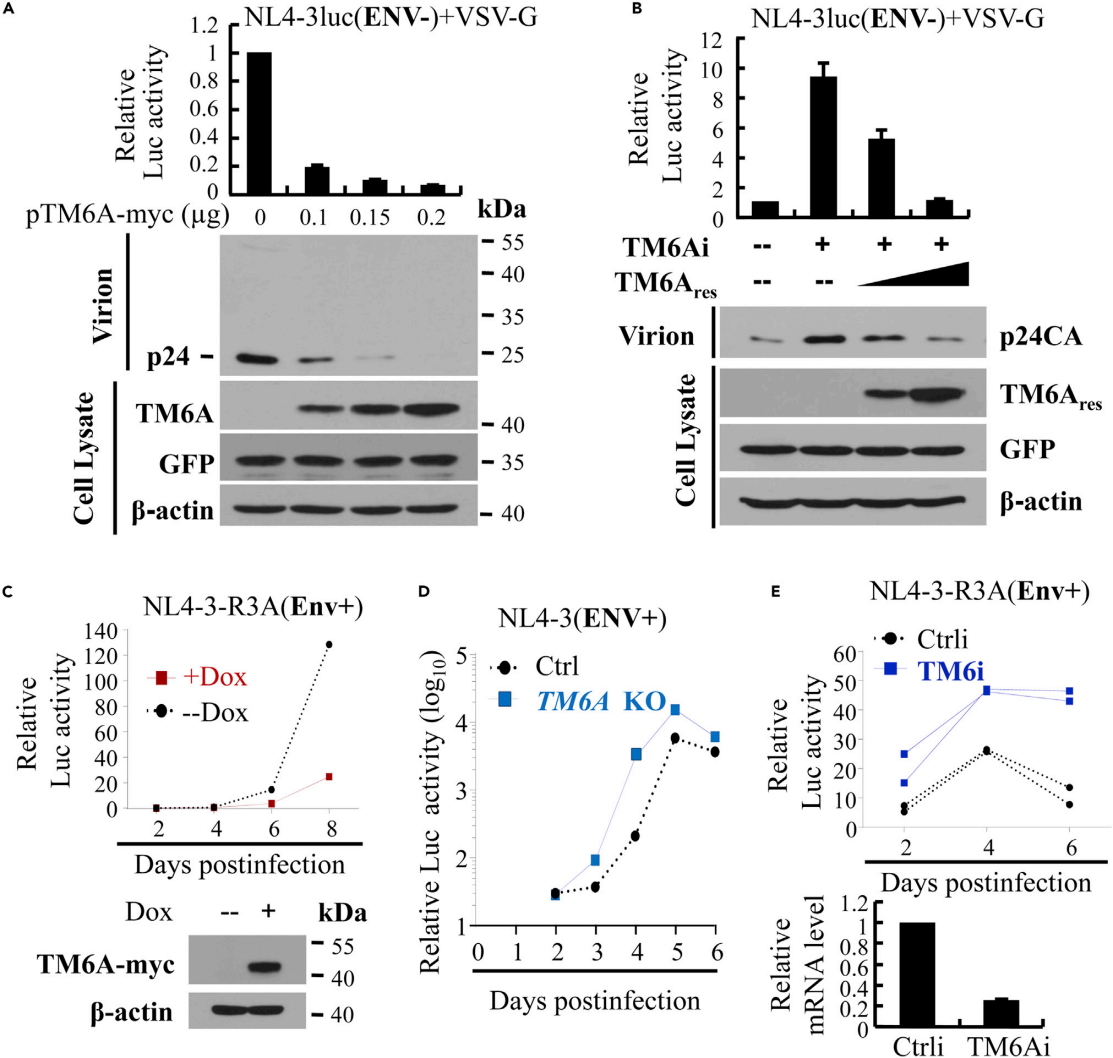
TMEM106A inhibits HIV-1 production (A and B) 293T cells were transfected with plasmids producing VSV-G pseudotyped NL4-3luc vector, together with a plasmid expressing myc-tagged TMEM106A (A) or a plasmid expressing the shRNA targeting TMEM106A and a rescue TMEM106A-expressing construct (B). A plasmid expressing GFP was included to serve as a control for transfection efficiency and sample handling. At 48 h post transfection, the producer cells were lysed for western analysis and the culture supernatants were collected to infect recipient cells. The relative luciferase activity in the recipient cells infected with the virus produced in the control cells was set as 1. Data presented are means ± SD of four independent experiments. TM6A, TMEM106A; Ctrli, control shRNA; TM6i, shRNA targeting TMEM106A. (C–E) Cells were infected with replication-competent HIV-1 virus. At the time points indicated, the relative virus titers in the culture supernatants were measured on the TZM-bl indicators cells, reflected by the relative luciferase activity. (C) THP1 cells expressing TMEM106A in a doxycycline-inducible manner was infected with the NL4-3-R3A virus. Data presented are representative of two independent experiments. (D) MT4 control cells (Ctrl) or *TMEM106A* knockout cells (*TM6A* KO) were infected with NL4-3 virus. Data presented are means ± SD of two independent measurements, representative of three independent experiments. (E) Human MDMs transfected with a control siRNA or an siRNA targeting TMEM106A were infected with NL4-3-R3A virus. TMEM106A mRNA levels in the MDMs were measured using β-actin mRNA as an internal control. This experiment was performed in duplicate and data presented are representative of two independent experiments.

To search for novel host antiviral factors, we screened a subset of human ISGs we recently identified (Zhang et al., 2018) for their ability to inhibit the production of HIV-1. The ISGs were each transiently co-transfected into 293T cells with plasmids producing the vesicular stomatitis virus G (VSV-G) pseudotyped HIV-1 vector NL4-3luc. In this reporter viral vector, Env expression is abolished by a nonsense mutation and a firefly luciferase reporter is inserted into the Nef coding sequence such that Nef is not expressed. The produced virus was used to infect recipient cells, and the luciferase activity in the recipient cells was measured to evaluate the effects of the ISG on viral production. Of the 113 ISGs we screened, TMEM106A displayed strong inhibitory activity (Table S1 and Figure 1A). TMEM106A expression reduced the luciferase activity in the recipient cells in a dose-dependent manner (Figure 1A, upper panel). TMEM106A expression also reduced the p24CA levels in the culture supernatant (Figure 1A, lower panels). In line with these results, downregulation of the endogenous TMEM106A enhanced the production of VSV-G pseudotyped NL4-3luc (Figure 1B).

To test its antiviral activity against the replication-competent HIV-1 virus, TMEM106A was expressed in THP-1 cells in a doxycycline-inducible manner. The THP-1 cells obtained from ATCC failed to support HIV-1 replication in our hands. However, with ectopic expression of human CD4 and SIV-Vpx, the engineered cells can support the replication of NL4-3-R3A, an HIV-1 virus in which the Env sequence of NL4-3 was replaced with that of HIV-1 strain R3A (Huang et al. unpublished results). The cells were challenged with NL4-3-R3A, and viral replication was monitored. TMEM106A expression inhibited the replication of the virus (Figure 1C). To investigate whether TMEM106A inhibits HIV-1 replication at the endogenous level, it was knocked out in MT4 cells. Two CRISPR single-guide RNAs (sgRNAs) targeting the genome of *TMEM106A* were expressed in the cells, and the knockout efficiency was confirmed by sequencing of the genomic DNA and by RT-qPCR (Figure S1). The cells were challenged with the replication-competent NL4-3 virus. In line with the above results, *TMEM106A* knockout enhanced the viral replication (Figure 1D). We also examined the antiviral activity of endogenous TMEM106A against NL4-3-R3A virus in primary human monocyte-derived macrophages (MDMs). Downregulation of TMEM106A increased the viral replication (Figure 1E). These results indicate that TMEM106A inhibits HIV-1 replication at the endogenous level or upon overexpression.

### TMEM106A inhibits HIV-1 virion release from the cell surface through its C-terminal intermolecular interaction

**Figure 2:**
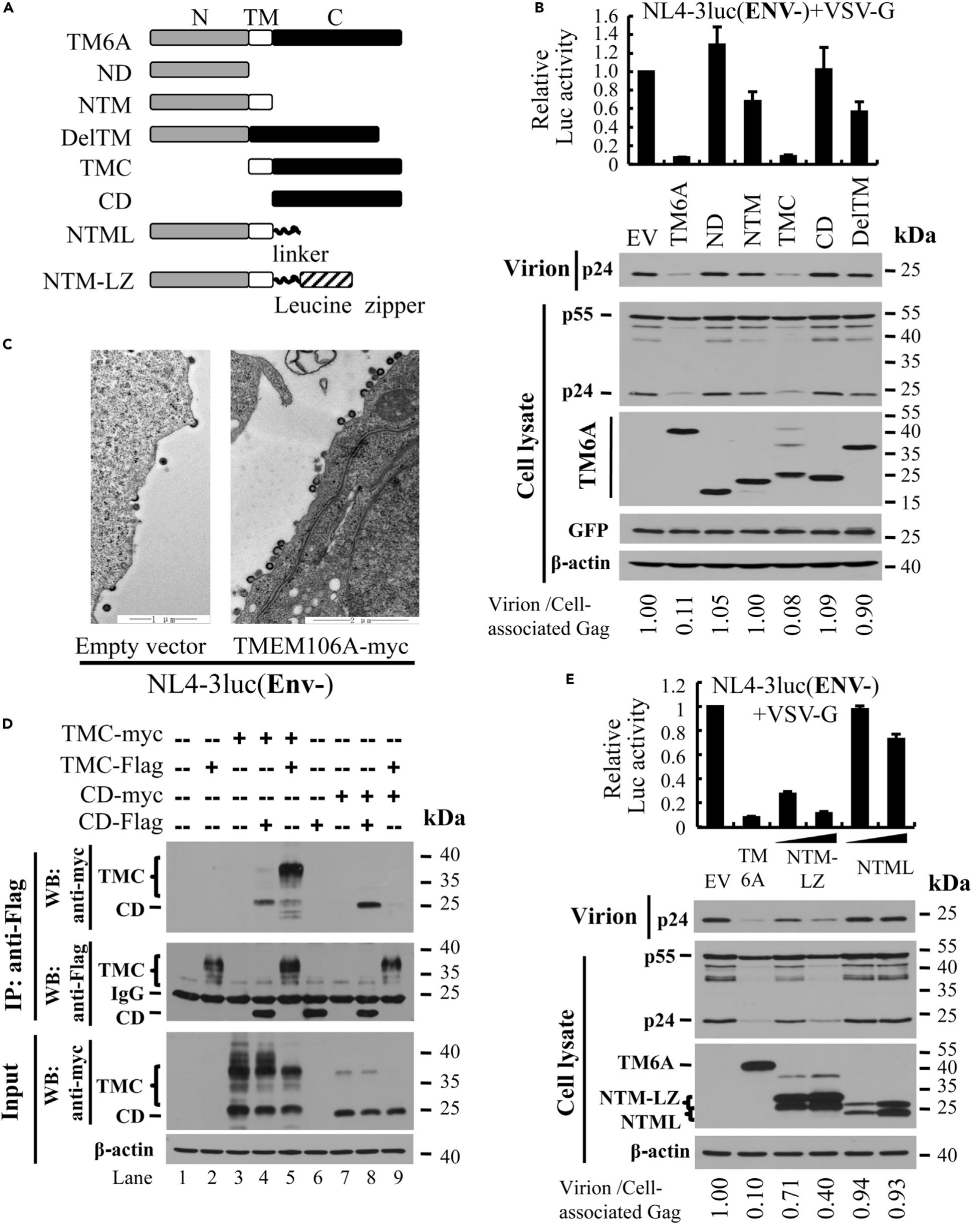
TMEM106A inhibits HIV-1 virion release from the cell surface through its intermolecular interaction of the C-terminal domain (A) Schematic representation of TMEM106A truncation mutants. (B) VSV-G pseudotyped HIV-1 vector-producing plasmids were transfected into 293T cells together with a plasmid expressing the myc-tagged truncation mutant. The antiviral activity of the mutant was analyzed as described in the legend to Figure 1A. Data presented are means ± SD of three independent experiments. (C) pNL4-3luc was transfected into 293T cells together with an empty vector or a plasmid expressing TMEM106A. At 36 h post transfection, cells were subjected to transmission electron microscopy analysis. (D) Myc-tagged and FLAG-tagged TMEM106A variants were transiently co-expressed in 293T cells. The cell lysates were immunoprecipitated with anti-FLAG affinity gel, followed by western analysis. (E) 293T cells were transfected with plasmids producing VSV-G pseudotyped NL4-3luc, together with a plasmid expressing the TMEM106A mutant indicated. The antiviral activity of the mutant was analyzed as described in the legend to Figure 1A. Data presented are means ± SD of three independent experiments. The viral protein levels were determined by measuring the band intensities of the proteins using the ImageJ software. Virion release efficiency was determined as the virion-associated p24 level divided by the total Gag protein level in the cell lysate. The relative virion release efficiency from the producer cells transfected with the empty vector was set as 1.

To probe the mechanism by which TMEM106A inhibits HIV-1 production, we first mapped the domains required for its antiviral activity. TMEM106A comprises an intracellular N-terminal domain (ND), a single transmembrane domain (TM), and an extracellular C-terminal domain (CD). Truncation mutants with a myc-tag at the C terminus (Figure 2A) were constructed and analyzed for their activities to inhibit the production of VSV-G pseudotyped NL4-3luc. The TMC mutant, which is composed of TM and CD, displayed antiviral activity comparable with that of the full-length protein (Figure 2B, upper panel). In contrast, overexpression of the other mutants had little effect on the production of the viral vector (Figure 2B, upper panel). The phenotypes of these mutants suggested that the membrane localization is critical for its antiviral activity. To support this notion, we analyzed the cell surface expression of these proteins. The proteins were transiently expressed in 293T cells, then the cells were surface stained with anti-myc antibody and analyzed by FACS. As expected, TMC was detected on the cell surface but CD and DelTM were not (Figure S2). Collectively, these results suggest that the C-terminal and TM domains are both essential for the antiviral activity of TMEM106A.

To investigate whether the reduced viral production was caused by TMEM106A inhibition of Gag expression or virion release, we analyzed the viral protein levels in the culture supernatant and the cell lysate. The culture supernatant was collected and subjected to western analysis. The producer cells were trypsinized to remove associated virion particles for better evaluation of the viral protein expression levels. In accordance with the above results, TMEM106A expression reduced the viral p24CA levels in the culture supernatant but had little effect on the p55Gag levels in the cell lysate (Figure 2B, lower panels). In addition, TMEM106A expression did not affect Gag association with the cellular membrane (Figure S3). These results suggested that TMEM106A inhibited the virion release. Noticeably, when HIV-1 virion release is inhibited, one would often expect to observe an increase in the cell-associated p24CA. However, here the p24CA level was reduced in the lysate of the cells expressing TMEM106A (Figure 2B, lower panels, compare lanes 1 and 2). One explanation is that the cells were trypsinized and the cell-associated virions were removed. In accordance with this speculation, we observed modestly increased cell-associated p24 levels when the cells were not trypsinized (Figure S4). Other possible explanations have been proposed previously (McNatt et al., 2009). One is that the virion particles trapped on the cell surface were destroyed. The possibility also exists that virion particles that can be successfully released from the cell surface are only a small portion of Gag proteins expressed in producer cells. Thus, whether they are retained to the cell surface has little effect on intracellular p24 levels and the amount of p24 levels in cell lysates would be determined by its intrinsic turnover rate.

To further demonstrate that TMEM106A inhibits virion release, we employed transmission electron microscopy. NL4-3luc virion particles were produced in 293T cells with or without ectopic expression of TMEM106A. Compared with the control cells, much more virion particles were retained on the surface of the cells overexpressing TMEM106A (Figure 2C), reminiscent of the phenotype of BST2. These results further indicate that TMEM106A trapped the virions on the cell surface.

Unlike BST2, TMEM106A has only one membrane anchoring domain, the transmembrane domain. To tether the virion particle to the cell surface, one part of the protein would need to be incorporated into the virion particle and another part would need to be on the cell surface. We speculated that intermolecular interactions of TMEM106A would allow it to function like BST2. To test this idea, we first analyzed whether the C-terminal domain of TMEM106A interacts with each other, considering that TMC was as active as the full-length protein. C-terminally FLAG-tagged TMC or CD was co-expressed with myc-tagged TMC or CD, and the interactions were evaluated by coimmunoprecipitation assays. Immunoprecipitation of TMC-FLAG coprecipitated TMC-myc (Figure 2D, lane 5). Immunoprecipitation of CD-FLAG coprecipitated CD-myc (Figure 2D, lane 8) and the putative unglycosylated form of TMC-myc (Figure 2D, lane 4). These results indicate that the C-terminal domain of TMEM106A interacts with each other.

To further show that the intermolecular interaction is critical for the antiviral activity, we replaced the C-terminal domain of TMEM106A with the leucine-zipper from the yeast transcriptional activator GCN4 (O'Shea et al., 1991). To provide steric flexibility of the leucine-zipper relative to the TM domain, a linker of 39 amino acids was inserted between the leucine-zipper and TM. The NTM-leucine-zipper protein (NTM-LZ) and a control protein that contains only the linker at the C terminus (NTML) were tested for their ability to inhibit the production of VSV-G pseudotyped NL4-3luc. NTM-LZ significantly inhibited the viral production (Figure 2E). In contrast, NTML had little effect (Figure 2E). These results further demonstrate that the intermolecular interaction of the C-terminal domain is important for the antiviral activity.

### HIV-1 envelope protein antagonizes TMEM106A

**Figure 3:**
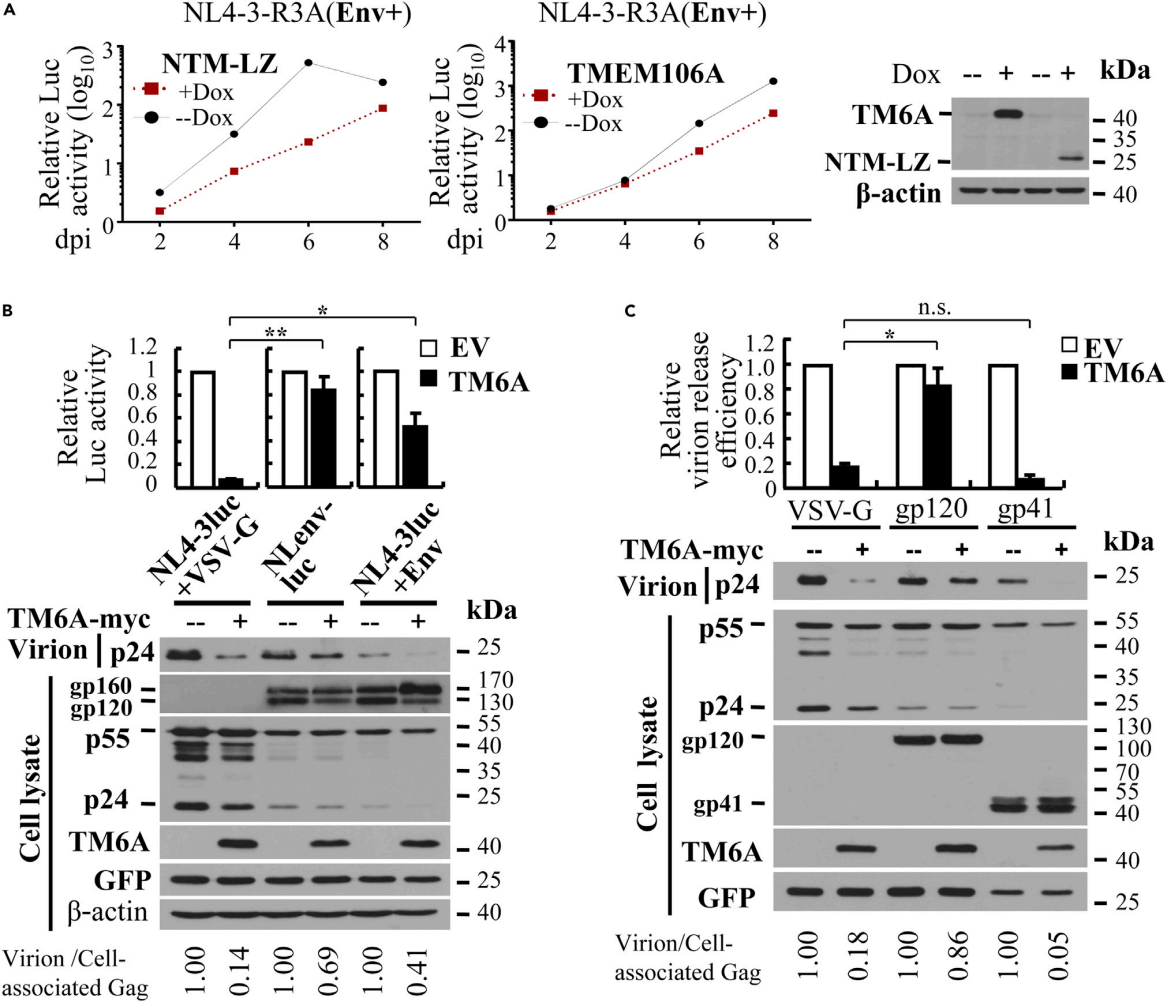
TMEM106A is antagonized by HIV-1 Env (A) THP1 cells expressing TMEM106A or NTM-LZ in a doxycycline-inducible manner were infected with NL4-3-R3A virus. The cells were mock treated or treated with doxycycline. At the time points indicated, relative virus titers in the supernatants were measured on TZM-bl cells. Data presented are representative of two independent experiments. (B) 293T cells were transfected with the virus-producing plasmids, with or without a plasmid expressing TMEM106A-myc. The antiviral activity of TMEM106A was analyzed as described in the legend to Figure 1A. Data presented are means ± SD of three independent experiments. (C) 293T cells were transfected with pNL4-3luc together with a plasmid expressing VSV-G, gp120, or gp41, with or without a plasmid expressing TMEM106A-myc. A plasmid expressing GFP was included to serve as a control for transfection efficiency and sample handling. At 48 h post transfection, the producer cells and culture supernatants were analyzed by western blotting. The relative virion release efficiency was determined as described above in the legend to Figure 2. Data presented are means ± SD of three independent experiments. ∗ denotes p < 0.05; ∗∗ denotes p < 0.01; n.s. denotes p > 0.05

To further confirm the antiviral activity of NTM-LZ, it was expressed in the THP-1 cells in a doxycycline-inducible manner and assayed for its ability to inhibit the replication of NL4-3-R3A. Indeed, overexpression of NTM-LZ inhibited the viral replication (Figure 3A). However, we noticed that NTM-LZ inhibited the viral replication more potently than TMEM106A, even though the TMEM106A protein was expressed at a higher level (Figure 3A). One possible explanation is that the intermolecular interaction mediated by the leucine-zipper was stronger than that mediated by the C-terminal domain of TMEM106A. One other possibility is that the antiviral activity of TMEM106A might be partially suppressed by the virus. Considering that multiple antagonisms have been reported for antiviral factors against HIV-1, we explored this possibility. We first compared the inhibitory activity of TMEM106A against the production of VSV-G pseudotyped NL4-3luc versus NLenv-luc. The difference between the two viruses is that the viral Envelope protein (Env) is expressed in NLenv-luc but not in NL4-3luc (Connor et al., 1995; Dang et al., 2006; Mu et al., 2015). Consistent with the above results in Figure 1A, TMEM106A dramatically inhibited the production of VSV-G pseudotyped NL4-3luc (Figure 3B). In comparison, its effect on the production of NLenv-luc was marginal (Figure 3B). These results suggested that the antiviral activity of TMEM106A might be antagonized by Env. To substantiate this notion, we analyzed whether ectopic expression of Env can suppress the antiviral activity of TMEM106A. Indeed, Env expression significantly relieved TMEM106A inhibition of the production of the viral vector (Figure 3B). Env (gp160) is expressed as a polyprotein and proteolytically cleaved into the surface subunit gp120 and transmembrane subunit gp41. When the two subunits were expressed separately, only gp120 suppressed the antiviral activity of TMEM106A against the production of NL4-3luc (Figure 3C). We also tested the antagonism activity of gp120 from other HIV-1 strains, including CI (R5-tropic) and 89.6 (dual tropic). They all displayed the antagonism activity to some extent (Figure S5). Collectively, these results indicate that HIV-1 gp120 antagonizes the antiviral activity of TMEM106A.

### HIV-1 gp120 interferes with the intermolecular interaction of the C-terminal domain of TMEM106A

**Figure 4:**
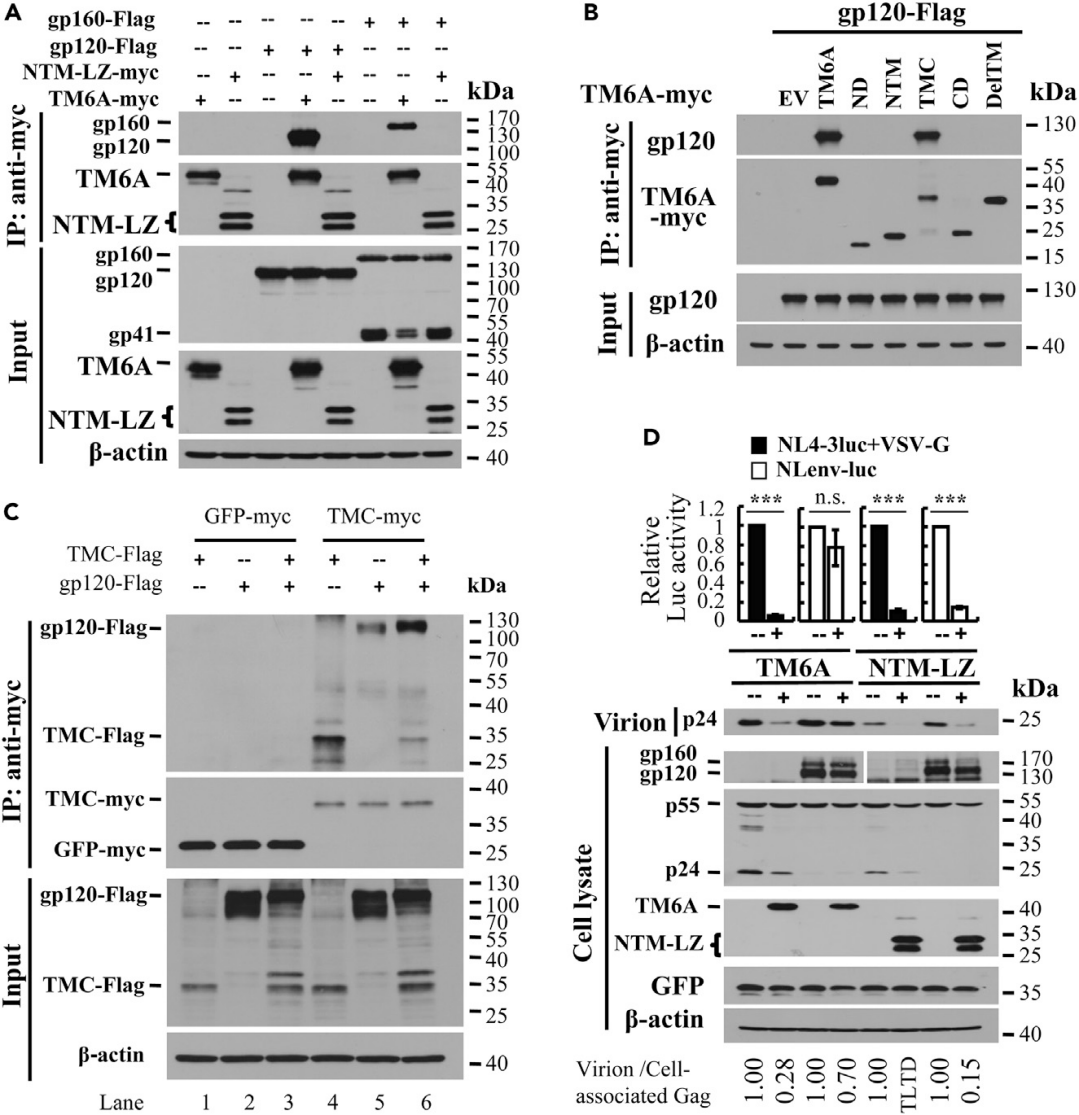
HIV-1 gp120 interferes with the intermolecular interaction of TMEM106A C-terminal domain (A and B) Proteins indicated were expressed in 293T cells. The cell lysates were immunoprecipitated, followed by western analysis. (C) Myc-tagged GFP or TMC was expressed in 293T cells. In a separate setting, FLAG-tagged TMC and gp120 were expressed in 293T cells. The cell lysates were mixed, immunoprecipitated, and subjected to western analysis. (D) Viruses indicated were produced with or without TMEM106A-myc or NTM-LZ-myc. The antiviral activity of TMEM106A and NTM-LZ was analyzed as described in the legend to Figure 1A. The relative virion release efficiency was determined as described above in the legend to Figure 2. TLTD, too low to detect. Data presented are means ± SD of four independent experiments. ∗∗∗denotes p < 0.001; n.s. denotes p > 0.05

We next explored how Env antagonized TMEM106A. Given that both gp120 and TMC are localized on the cell surface, we hypothesized that gp120 might interact with the extracellular domain of TMEM106A and thereby interfere with the intermolecular interaction of TMEM106A. We first tested the interaction between TMEM106A and gp120 by coimmunoprecipitation assays. C-terminally FLAG-tagged gp120 or gp160 was co-expressed with myc-tagged TMEM106A or NTM-LZ in 293T cells. gp160 was partially processed into gp120 and gp41. Since gp160 was FLAG-tagged at the C terminus, only the processing product gp41 and the unprocessed gp160 could be detected by the anti-FLAG antibody. Immunoprecipitation of TMEM106A coprecipitated gp120 and gp160 (Figure 4A), indicating that TMEM106A indeed interacts with gp120. In contrast, immunoprecipitation of NTM-LZ failed to do so (Figure 4A). TMEM106A did not interact with gp41 (Figure S6B, lane 8), consistent with the above results in Figure 3C that gp41 did not antagonize TMEM106A. Confocal microscopy analysis results revealed that TMEM106A colocalized with gp160 on the plasma membrane, as well as in the cytoplasm (Figure S6A), consistent with the notion that TMEM106A interacts with gp120. TMEM106A did not interact with VSV-G (Figure S6C), consistent with the above results in Figures 3B and 3C that VSV-G did not antagonize TMEM106A. gp120 interacted with TMC as well as the full-length TMEM106A protein (Figure 4B), indicating that the C-terminal domain is the gp120-binding domain. To test whether gp120 interferes with the intermolecular interaction of TMEM106A, the interaction between FLAG-tagged TMC and myc-tagged TMC was analyzed in the absence or presence of gp120. Indeed, gp120 significantly reduced the intermolecular interaction of TMC (Figure 4C, compare lanes 4 and 6). Since NTM-LZ did not interact with gp120, gp120 should not antagonize NTM-LZ. As expected, NTM-LZ inhibited the production of both VSV-G pseudotyped NL4-3luc and NLenv-luc (Figure 4D). These results indicate that gp120, but not gp41 or VSV-G, interacts with TMEM106A and interferes with the intermolecular interaction to suppress its antiviral activity. Further analysis revealed that overexpression of gp120 did not affect TMEM106A protein levels on the cell surface (Figure S7).

### TMEM106A is incorporated into HIV-1 virion particles

**Figure 5:**
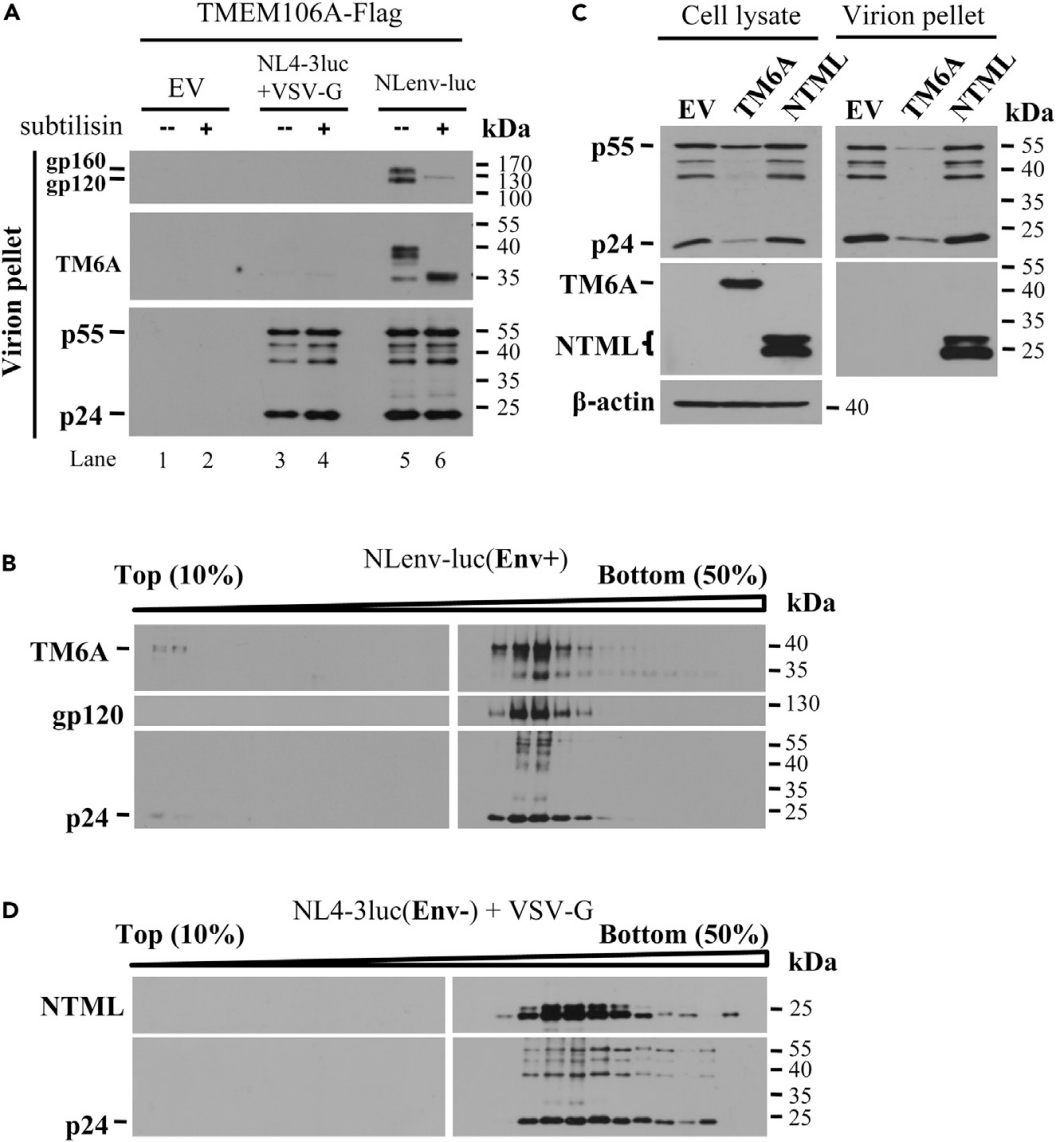
TMEM106A is incorporated into HIV-1 virion particles (A and B) Virion particles indicated were produced in 293T cells in the presence of TMEM106A-FLAG. (A) Comparable amounts of virion particles were pelleted by ultracentrifugation, mock treated or treated with subtilisin, ultracentrifuged again, and subjected to western blotting analysis. (B) Virions produced from NLenv-luc without subtilisin treatment were applied to 10%–50% linear sucrose gradient centrifugation. Fractions were collected and analyzed by western blotting. (C and D) 293T cells were transfected with plasmids producing VSV-G pseudotyped NL4-3luc, together with a plasmid expressing myc-tagged TMEM106A or NTML. At 48 h post transfection, cells were lysed and culture supernatants were collected. (C) The culture supernatants were ultracentrifuged through 25% sucrose cushion. Protein levels in producer cells and ultracentrifugation pellets were analyzed by western blotting. (D) Virions produced in the presence of NTML were applied to 10%–50% linear sucrose gradient centrifugation. Fractions were collected and analyzed by western blotting

For TMEM106A to inhibit the release of virion particles from the cell surface, one would expect that it needs to be incorporated into virion particles. To test this idea, VSV-G pseudotyped NL4-3luc and NLenv-luc virions were produced in 293T cells in the presence of FLAG-tagged TMEM106A. The virion-containing culture supernatants were purified by ultracentrifugation through sucrose cushion, treated with protease subtilisin, centrifuged again, and analyzed by western blotting. As expected, the yield of the VSV-G pseudotyped virions was much lower than that of the NLenv-luc virions (data not shown). For easy comparison, comparable amounts of virion particles were used for analysis. TMEM106A was detected in the virion pellet of NLenv-luc but not in the pellet of VSV-G pseudotyped NL4-3luc (Figure 5A). Treatment of the virions with subtilisin removed Env and TMEM106A but did not affect the Gag proteins (Figure 5A). To further demonstrate that TMEM106A was incorporated into HIV-1 virions, the purified NLenv-luc virions were subjected to 10%–50% linear sucrose gradient centrifugation. Analysis of gradient fractions revealed that TMEM106A was detected in the virion-containing fractions (Figure 5B). Furthermore, the NTML mutant, which did not inhibit virus production, was incorporated into VSV-G pseudotyped NL4-3luc virions (Figure 5C). NTML also co-migrated with the virion particles in the sucrose gradient centrifugation assay (Figure 5D). Collectively, these results indicate that TMEM106A can be incorporated into virion particles.

### TMEM106A orthologs inhibit the replication of multiple enveloped viruses

**Figure 6:**
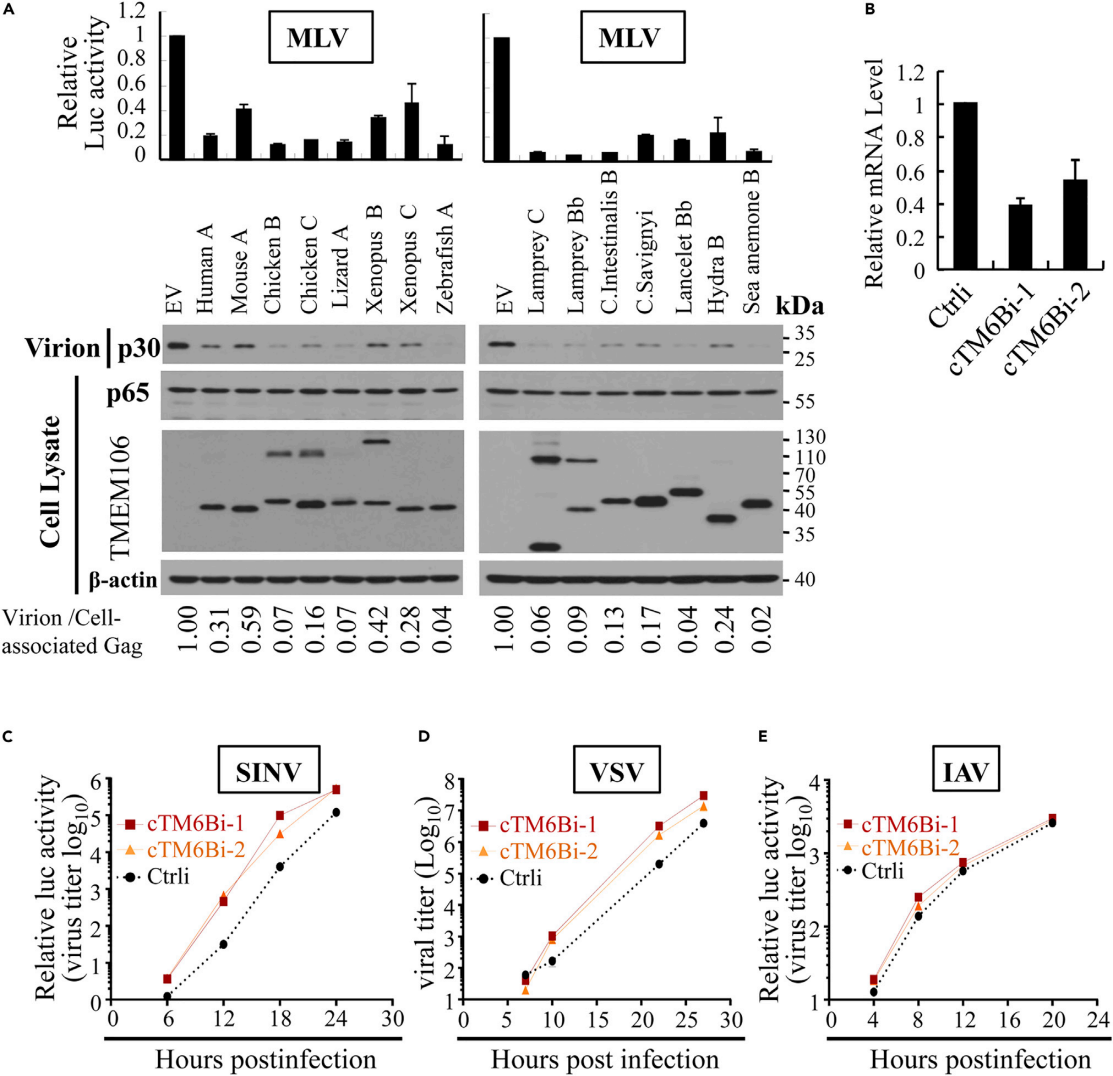
TMEM106 orthologs inhibit the production of multiple enveloped viruses (A) 293T cells were transfected with pNCA and pMLV-luc to produce infectious MLV-luc, together with an empty vector (EV) or a plasmid expressing myc-tagged TMEM106 indicated. At 48 h post transfection, the culture supernatants were used to infect Rat2 cells. At 48 h post infection, the luciferase activity in the Rat2 cells were measured, which reflects the relative titer of the produced virus. The relative virion release efficiency was determined as described above in the legend to Figure 2. Data presented are means ± SD of two independent experiments. (B–E) DF-1 cells were transfected with a control siRNA or siRNAs targeting different sites of chicken TMEM106B (cTMEM106B). (B) The cTMEM106B mRNA levels were measured by RT-qPCR using GAPDH mRNA as an internal control. (C) The cells were infected with SINV-nLuc. At the time points indicated, nLuc activity in the cells was measured. Data presented are representative of three independent experiments. (D) The cells were infected with VSV-GFP. At the time points indicated, the virus titer in the supernatant was measured by plaque assay. Data presented are means ± SD of three independent measurements, representative of three independent experiments. (E) The cells were infected with IAV-GLuc. At the time points indicated, GLuc activity in the supernatant was measured. Data presented are representative of three independent experiments.

The foregoing results revealed that TMEM106A is incorporated into HIV-1 virion particles and that through the intermolecular interaction of the C-terminal domain it traps the virion particles to the cell surface. Such mechanism of action suggested that TMEM106A might also inhibit the release of other enveloped viruses. To test this idea, we first analyzed the antiviral activity of TMEM106A against murine leukemia virus (MLV). To facilitate the quantification of MLV production, the wild-type MLV proviral DNA pNCA and luciferase-expressing vector pMLV-luc were co-transfected into 293T cells to produce infectious MLV-luc. The produced virus was used to infect recipient cells, and the luciferase activity in the recipient cells served as an indicator of the viral production. TMEM106A overexpression inhibited MLV production, without affecting the p65Gag expression level in the producer cells (Figure 6A). To investigate whether TMEM106A inhibits MLV production at the endogenous level, it was downregulated in HEK293 cells. An shRNA targeting the 3′ UTR of TMEM106A mRNA was validated for its ability to downregulate TMEM106A expression (Figures S8A and S8B). In line with the above results, downregulation of TMEM106A enhanced the production of the virus (Figure S8C).

Human TMEM106A has two paralogues, TMEM106B and TMEM106C (Figure S9). Sequence analysis and previous studies revealed that, unlike TMEM106A, which is localized on the plasma membrane with the C-terminal domain outside (Dai et al., 2015; Xu et al., 2014 and Figure S10), TMEM106B is localized in the endosomes and lysosomes (Brady et al., 2013; Busch et al., 2016; Chen-Plotkin et al., 2012; Lang et al., 2012; Nicholson et al., 2013; Stagi et al., 2014) and TMEM106C has two transmembrane regions with both N- and C-terminal domains in the cytoplasm (Figure S10). To test their antiviral activity, they were co-transfected with the MLV-luc-producing plasmids into 293T cells. TMEM106A inhibited the viral production, whereas TMEM106B failed to do so (Figure S11). TMEM106C displayed antiviral activity comparable with that of TMEM106A (Figure S11) (see below for further discussion).

TMEM106 genes can be found in species as ancient as lancelet, hydra, and sea anemone. Sequence analysis predicted that they are all type II transmembrane proteins like human TMEM106A (Figure S10). It was confirmed by FACS analysis that the C-terminal domains of these proteins are the extracellular domains (Figure S12). We thus consider they are the orthologs of human TMEM106A, although they are named TMEM106B or 106C in some species. To explore whether TMEM106A is an evolutionarily conserved antiviral factor, we tested the antiviral activity against MLV of some TMEM106A orthologs from representative species, including mouse, chicken, lizard, xenopus, zebrafish, lamprey, *Ciona intestinalis*, *Ciona savignyi*, lancelet, hydra, and sea anemone. Overexpression of these proteins inhibited the production of MLV without obviously affecting the p65Gag level in the producer cells (Figure 6A). Consistently, when some representatives were expressed in Rat2 cells, they inhibited the replication of MLV (Figure S13). These results suggested that TMEM106A is an evolutionarily conserved antiviral factor against MLV.

To explore whether TMEM106A is also active against other enveloped viruses, the expression of the endogenous chicken TMEM106B (cTMEM106B) was downregulated in DF-1 cells using two siRNAs (Figure 6B). As described above, cTMEM106B is a type II transmembrane protein localized on the plasma membrane and thus considered the ortholog of human TMEM106A. The cells were challenged with Sindbis virus (SINV), vesicular stomatitis virus (VSV), or influenza A virus (IAV), which are enveloped RNA viruses in the Togaviridae, Rhabdoviridae, and Orthomyxoviridae families, respectively. Downregulation of cTMEM106B significantly increased the replication of SINV (Figure 6C) and VSV (Figure 6D). However, downregulation of cTMEM106B had little effect on IAV replication (Figure 6E). These results indicate that the effect of cTMEM106B downregulation on SINV and VSV replication was not likely caused by nonspecific alteration of the cells and suggest that IAV may have an antagonistic mechanism (see below for further discussion). Collectively, these results support the notion that TMEM106A is an evolutionarily conserved antiviral factor against enveloped viruses.

## Discussion

**Figure 7:**
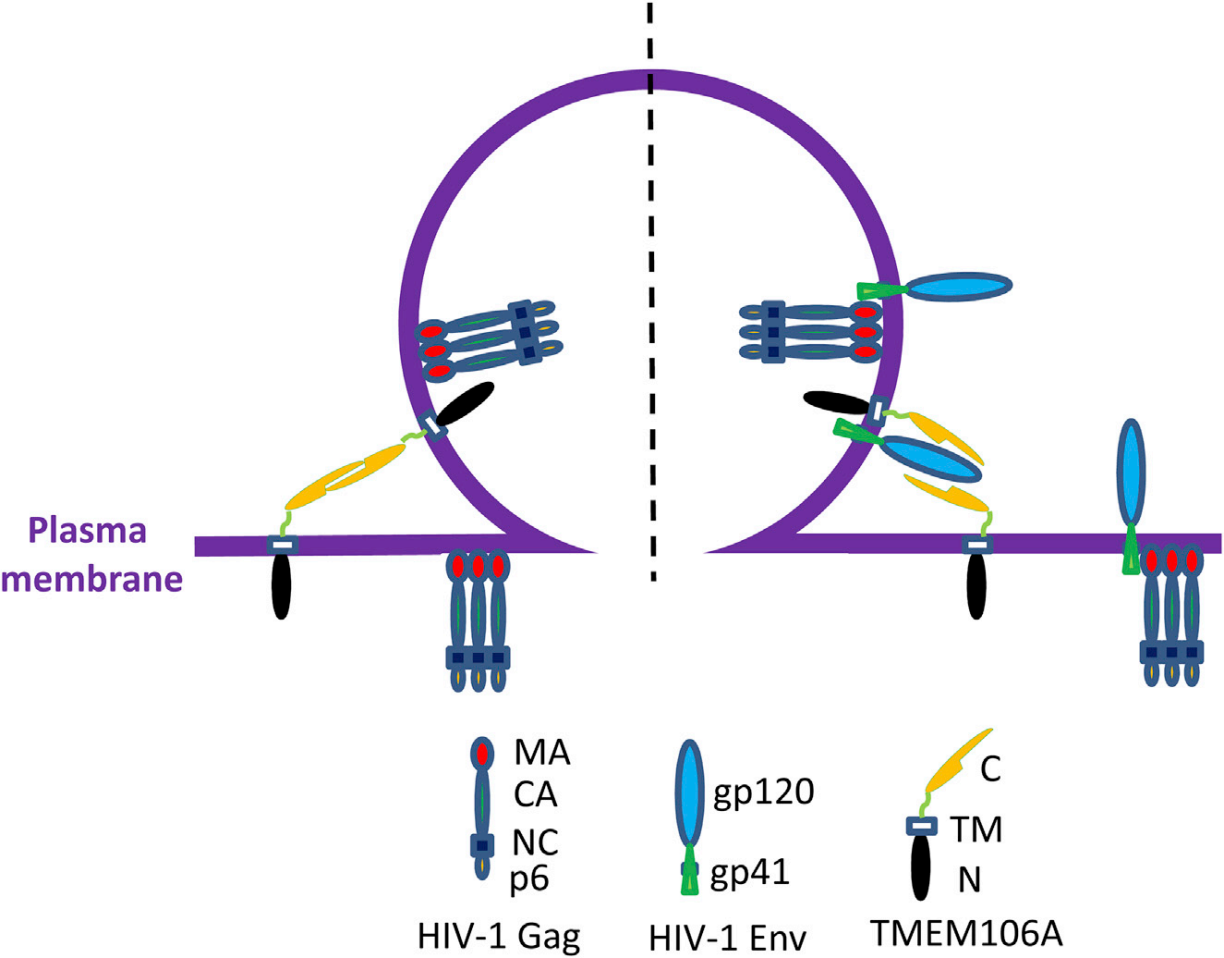
Working model for interactions between TMEM106A and HIV-1 TMEM106A is incorporated into HIV-1 virions. In the absence of HIV-1 Env (left), the C-terminal domain of the TMEM106A on the cell surface interacts with the C-terminal domain of TMEM106A on the virion surface. This intermolecular interaction tethers the virion to the cell surface. In the presence of HIV-1 Env (right), gp120 interferes with the intermolecular interaction. As a result, the inhibitory activity of TMEM106A against virion release is partially suppressed. The antagonizing Env can be localized either on the virion surface or on the cell surface.

In this report, we identified human TMEM106A as a host antiviral factor that inhibited the release of HIV-1 from the cell surface. We provided evidence showing that TMEM106A also inhibits the replication of other enveloped viruses. Based on the results in this report, we propose a working model for TMEM106A to inhibit HIV-1 virion release (Figure 7). TMEM106A is expressed on the cell surface, and some molecules are incorporated into virion particles. The extracellular C-terminal domain on the virion surface interacts with that on the cell surface, tethering the virion particle to the cell surface. Replacement of the C-terminal domain of TMEM106A with a leucine-zipper rendered the protein more active than the wild-type protein (Figures 3A and 4D), highlighting the importance of the C-terminal intermolecular interactions. Human TMEM106B is localized on the membrane of lysosomes and endosomes and did not display any antiviral activity (Figure S11). These results are consistent with the notion that the TMEM106 proteins inhibit virion release from the cell surface. Human TMEM106C has two transmembrane domains and displayed antiviral activity comparable with that of TMEM106A. It could use the two transmembrane domains to tether the virion particle to the cell surface, like BST2. Its mechanism of action needs to be further investigated.

It is not clear how TMEM106A is incorporated into virions. Previous studies suggest that localization of transmembrane proteins in the virus budding sites could trigger the package of target proteins into virions. BST2 was reported to localize on cholesterol-enriched lipid rafts at the plasma membrane (Kupzig et al., 2003), which are the budding sites of many enveloped viruses (Nguyen and Hildreth, 2000; Panchal et al., 2003). The serine incorporator 5 (SERINC5), a transmembrane protein that inhibits viral infectivity, was suggested to be incorporated into virions by localization to the budding sites (Firrito et al., 2018). Confocal microscopy analysis revealed that TMEM106A colocalized with CD230, a marker for lipid raft (Figure S14A). In addition, TMEM106A seemed to partially colocalize with HIV-1 Gag in the cytoplasm, as well as on the plasma membrane (Figure S14B). These results suggest that TMEM106A might also be incorporated into virion particles at the virus budding sites.

HIV-1 Env interacts with TMEM106A through the gp120 domain and thereby interferes with the intermolecular interactions of TMEM106A, suppressing its antiviral activity. Since the antagonism is mediated by Env competitive interaction with TMEM106A, it would not be expected to be complete, which is consistent with the results that TMEM106A overexpression inhibited (Figures 1C and 3A) and downregulation enhanced (Figures 1D and 1E) the replication of wild-type HIV-1 virus. Since HIV-1 Env did not interact with the leucine-zipper (Figure 4A), it failed to antagonize NTM-LZ (Figures 3A and 4D). Consistently, NTM-LZ displayed higher antiviral activity than TMEM106A against the replication of HIV-1 (Figure 3A). The difference in the antiviral activity between NTM-LZ and TMEM106A supports the notion that the Env antagonism functions in HIV-1 replication.

In addition to HIV-1, TMEM106A overexpression inhibited the replication of MLV (Figure S13). Furthermore, downregulation of the TMEM106A ortholog cTMEM106B promoted the replication of SINV and VSV (Figures 6C and 6D). These results indicate that TMEM106A is a broad-spectrum antiviral factor against enveloped viruses. Downregulation of cTMEM106B had little effect on the replication of IAV (Figure 6E), suggesting that IAV may have a mechanism to counteract the antiviral function of cTMEM106B. Multiple IAV antagonisms have been reported. For example, NS1 antagonizes the short form of ZAP (ZAPS) through suppressing its binding to target viral mRNAs (Tang et al., 2017). NA, NS1, and M2 have been reported to counteract the antiviral activity of BST2 (Hu et al., 2017; Leyva-Grado et al., 2014; Watanabe et al., 2011; Yondola et al., 2011). How IAV antagonizes TMEM106A awaits further investigation.

The TMEM106A orthologs tested in this report all displayed significant antiviral activity against the production of MLV, suggesting that it is an evolutionarily conserved antiviral factor, although further investigation is needed to prove this. The cellular function of TMEM106A is not very clear, although it is implicated as a tumor suppressor in some cancers (Liu and Zhu, 2018; Wu et al., 2017; Xu et al., 2014). It is not clear whether the putative evolutionary conservation is driven by its cellular function or by its antiviral activity. Nonetheless, the conserved antiviral activity highlights its importance in the antiviral defense system.

In summary, we identified TMEM106A as an evolutionarily conserved antiviral factor that inhibits virion release from the cell surface of multiple enveloped viruses. These results indicate the hosts have evolved multiple mechanisms targeting the virion release process shared by enveloped viruses. These results also suggest that some antiviral factors exist in lower vertebrates and invertebrates and are conserved during evolution.

## STAR★Methods

### Key resources table

**Table:** 

REAGENT or RESOURCE	SOURCE	IDENTIFIER
**Antibodies**		

Mouse monoclonal Flag-specific antibody M2	Sigma-Aldrich	Cat# F3165-5MG; RRID: AB_259529
Rabbit polyclonal DYKDDDDK Tag antibody	Cell Signaling Technology	Cat# 2368; RRID: AB_2217020
Mouse monoclonal myc-specific antibody 9E10	Santa Cruz Biotechnology	Cat# SC-40; RRID: AB_627268
Anti-Mouse IgG (whole molecule)-TRITC antibody	Sigma-Aldrich	Cat# T5393; RRID:AB_261699
Rabbit monoclonal Myc-Tag antibody	Cell Signaling Technology	Cat# 2278; RRID:AB_490778
Mouse monoclonal β-actin-specific antibody	Sigma-Aldrich	Cat# a5316; RRID: AB_476743
Goat polyclonal HIV1 gp120 antibody	Abcam	Cat# ab21179; RRID:AB_732949
Mouse monoclonal p24-specific antibody P5F1	(Liu et al., 2007)	N/A
Rabbit polyclonal p30 antibody	(Mai and Gao, 2010)	N/A
Anti-Mouse IgG (H + L), HRP Conjugate	Promega	Cat# w4021; RRID: AB_430834
Peroxidase-Conjugated Goat anti-Rabbit IgG(H + L)	ZSGB-Bio	Cat# ZB-2301; RRID: AB_2747412
APC Goat anti-mouse IgG	BioLegend	Cat# 405308; RRID:AB_315011

**Bacterial and Virus Strains**		

Trans10 Chemically Competent Cell	Transgen Biotech	Cat# CD101-01
IAV-Gluc	(Pan et al., 2013)	N/A
SINV-nLuc	(Wang et al., 2016)	N/A
VSV-GFP	(Dalton and Rose, 2001)	N/A

**Chemicals, Peptides, and Recombinant Proteins**		

Doxycycline	Sigma-Aldrich	Cat# D9891-1G
Recombinant Human Macrophage Colony Stimulating Factor	Sangon Biotech	Cat# C600148-0002
Lipofectamine™ 2000 Transfection Reagent	ThermoFisher Scientific	Cat# 11668019
TRIzol™ Reagent	ThermoFisher Scientific	Cat# 15596018
^32^P-dTTP	Perkin Elmer	NEG005H250UC
Xpregen Transfection Reagent	Beijing Yu-Feng Biotechnology	Cat# ND01
DAPI Staining Solution	Beyotime biotechnology	Cat# C-1005

**Critical Commercial Assays**		

Luciferase Assay System	Promega	Cat# E1501
Passive Lysis 5X Buffer	Promega	Cat# E1941
Dual-Luciferase® Reporter Assay System	Promega	Cat# E1910
CelLytic™ M	Sigma-Aldrich	Cat# C2978
SuperReal PreMix Plus (SYBR Green)	Tiangen	Cat# Fp205-02
CD14 Microbeads	MACS Miltenyi Biotec	130-050-201
Anti-c-Myc Agarose Affinity Gel antibody produced in rabbit	Sigma-Aldrich	Cat# A7470
ANTI-FLAG® M2 Affinity Gel	Sigma-Aldrich	Cat# A2220

**Experimental Models: Cell Lines**		

293T cell	ATCC	CRL-11268
HEK293	ATCC	CRL-1573
293A	Invitrogen	LSR70507
Rat2	ATCC	CRL-1764
DF1	ATCC	CRL-12203
BHK-21(C13)	ATCC	CCL-10
HeLa	ATCC	CCL-2
HeLa-CD4-CCR5	(Deng et al., 1996)	N/A
TZM-bl cell	NIH AIDS Reagent Program	8129
THP-1 cell	ATCC	TIB-202
MT4 cell	NIH AIDS Reagent Program	120
THP1-TM6A-myc	This study	N/A
THP1-NTM-LZ-myc	This study	N/A
MT4-*Ctrl*	This study	N/A
MT4-*TM6A* KO	This study	N/A
Human MDM	This study	N/A
293T-TripZ-TM6A	This study	N/A
Rat2- EasiLV-Ev	This study	N/A
Rat2- EasiLV-mouseTM6A	This study	N/A
Rat2- EasiLV-chickenTM6B	This study	N/A
Rat2- EasiLV-zebrafishTM6A	This study	N/A

**Oligonucleotides**		

Ctrli: 5′-GCGCGCTTTGTAGGATTCGTT-3′ (Control shRNA sequence)	This study	N/A
TM6Ai: 5′-TGTGTGTATGAAGTTAACT-3′ (shRNA targeting sequence)	This study	N/A
*Ctrl*-sgRNA: 5′-AAATGTGAGATCAGAGTAAT-3′ (Control sgRNA sequence)	This study	N/A
*TM6A*-sgRNA1: 5′-GGTAAGACGTTTTCCCAGCT-3′ (sgRNA targeting sequence)	This study	N/A
*TM6A*-sgRNA2: 5′-GGTGGCTCTCATTCCCTATG-3′ (sgRNA targeting sequence)	This study	N/A
Ctrli: 5′-UUCUCCGAACGUGUCACGUTT-3′ (Control siRNA sequence)	This study	N/A
TM6Ai: 5′-GGGAGAAGUGGUGACAGAATT-3′ (siRNA targeting sequence)	This study	N/A
cCtrli: 5′-UUCUCCGAACGUGUCACGUTT-3′ (control siRNA sequence)	This study	N/A
cTM6Bi-1: 5′-CCAGGGAACGGGAAGAAUUTT-3′ (siRNA targeting sequence)	This study	N/A
cTM6Bi-2: 5′-GCAUGUAAUGCAUAGGAAATT-3′ (siRNA targeting sequence)	This study	N/A
qTM6A-F: 5′-CTGCCACCCATCTGATAGTAAG-3′	This study	N/A
qTM6A-R: 5′-GGAACCAGGGACAGTCAATAA-3′	This study	N/A
qGAPDH-F: 5′-TCGGAGTCAACGGATTTG-3′	This study	N/A
qGAPDH-R: 5′-GCATCGCCCCACTTGATT-3′	This study	N/A
qACTIN-F: 5′-CACCATTGGCAATGAGCGGTTC-3′	This study	N/A
qACTIN-R: 5′-AGGTCTTTGCGGATGTCCACGT-3′	This study	N/A
Genotype-F: 5′-GCCAGTCTTGAGAACTTCAAACC-3′	This study	N/A
Genotype-R: 5′-CAGGGGCAATGGCTTGTGA-3′	This study	N/A
qKO*TM6A*-F: 5′-GTGCCTTGTGAAGGAACTGCTG-3′	This study	N/A
qKO*TM6A*-R: 5′-CCATAGGGAATGAGAGCCACCA-3′	This study	N/A
qcTM6B-F: 5′-TAGTGTGGAATGACTGCTCTTG-3′	This study	N/A
qcTM6B-R: 5′-AGGCCACCATGTTTCCTATG-3′	This study	N/A
qcGAPDH-F: 5′-CAGAACATCATCCCAGCGTC-3′	This study	N/A
qcGAPDH-R: 5′-CAGGTCAGGTCAACAACAGAG′	This study	N/A

**Recombinant DNA**		

pNL4-3-R3A	(Zhang et al., 2011)	N/A
pNL4-3	NIH AIDS Reagent Program	Cat# 114
pNLenv-luc	(Dang et al., 2006)	N/A
pNL4-3luc	(Connor et al., 1995)	N/A
pEasiLV-MCS	(Goujon et al., 2013)	N/A
pEasiLV-mouseTM6A-3myc	This study	N/A
pEasiLV-chickenTM6B-3myc	This study	N/A
pEasiLV-zebrafishTM6A-3myc	This study	N/A
pcDNA4-humanTM6A-3myc	This study	N/A
pcDNA4-humanTM6B-3myc	This study	N/A
pcDNA4-humanTM6C-3myc	This study	N/A
pcDNA4-mouseTM6A-3myc	This study	N/A
pcDNA4-chickenTM6B-3myc	This study	N/A
pcDNA4-chickenTM6C-3myc	This study	N/A
pcDNA4-lizardTM6A-3myc	This study	N/A
pcDNA4-xenopusTM6B-3myc	This study	N/A
pcDNA4-xenopusTM6C-3myc	This study	N/A
pcDNA4-zebrafishTM6A-3myc	This study	N/A
pcDNA4-lampreyTM6C-3myc	This study	N/A
pcDNA4-lampreyTM6Bb-3myc	This study	N/A
pcDNA4-c.intestinalisTM6B-3myc	This study	N/A
pcDNA4-c.savignyiTM6B-3myc	This study	N/A
pcDNA4-lanceletTM6Bb-3myc	This study	N/A
pcDNA4-hydraTM6B-3myc	This study	N/A
pcDNA4- sea anemoneTM6B-3myc	This study	N/A
pcDNA4- NTM-LZ-3myc	This study	N/A
pcDNA4- NTML-3myc	This study	N/A
pcDNA4-ND-3myc	This study	N/A
pcDNA4-NTM-3myc	This study	N/A
pcDNA4-DelTM-3myc	This study	N/A
pcDNA4-TMC-3myc	This study	N/A
pcDNA4-CD-3myc	This study	N/A
pcDNA4-TMC-2flag	This study	N/A
pcDNA4-CD-2flag	This study	N/A
pcDNA-TM6A-2flag	This study	N/A
pEF1α-gp160	This study	N/A
pEF1α-gp160-2flag	This study	N/A
pEF1α-gp120(NL4-3)-2flag	This study	N/A
pEF1α-gp120(CI)-2flag	This study	N/A
pEF1α-gp120(89.6)-2flag	This study	N/A
pEF1α-gp41-2flag	This study	N/A
pcDNA4-VSV-G-2flag	This study	N/A
pcDNA4-IRES-mCherry	This study	N/A
pcDNA4-gp160-IRES-mCherry	This study	N/A
pcDNA4-gp120-IRES-mCherry	This study	N/A
pcDNA4-humanTM6A-mcherry	This study	N/A
pN1-TM6A-EGFP	This study	N/A
pN1-gp160-EGFP	This study	N/A
pHIV-Gag-iGFP-ΔEnv	(Hubner et al., 2007; Micsenyi et al., 2013)	N/A
pcDNA4-CD230-3myc	This study	N/A
pLentiCRISPR v.2-sgCtrl	This study	N/A
pLentiCRISPR v.2-sgTM6A-1	This study	N/A
pLentiCRISPR v.2-sgTM6A-2	This study	N/A
pSuper-Retro-Ctrli	This study	N/A
pSuper-Retro-TM6Ai	This study	N/A
pMLV-luc	(Gao et al., 2002)	N/A
pHIT60	(Gao et al., 2002)	N/A
pNCA	(Colicelli and Goff, 1988)	N/A
pTripZ	Horizon	Cat# RHS4750
pTripZ-TM6A-3myc	This study	N/A
pTripZ-NTM-LZ-3myc	This study	N/A

**Software and Algorithms**		

GraphPad Prism 8	GraphPad software	N/A
ImageJ	National Institutes of Health	https://imagej.nih.gov/ij
MEGA7	MEGA software	https://www.megasoftware.net
GeneDoc	GeneDoc software	http://nrbsc.org/gfx/genedoc

### Resource availability

#### Lead contact

Further information and requests for resources and reagents should be directed to and will be fulfilled by the lead contact, Dr. Guangxia Gao (gaogx@moon.ibp.ac.cn).

#### Materials availability

This study did not generate new unique reagents.

### Experimental model and subject details

#### Cell lines

293T (ATCC CRL-11268), HEK293 (ATCC CRL-1573), 293A (Invitrogen, LSR70507), TZM-bl (NIH AIDS Reagent Program 8129), BHK-21 (ATCC CCL-10), Rat2 (ATCC CRL-1764), HeLa (ATCC CCL-2) and HeLa-CD4-CCR5 (Deng et al., 1996) cells were maintained in Dulbecco's modified Eagle's medium (DMEM, Invitrogen) supplemented with 10% fetal bovine serum (FBS, Gibco), penicillin and streptomycin at 37°C, 5% CO2. DF-1 cells (CRL-12203) were maintained in DMEM supplemented with 10% FBS, penicillin and streptomycin at 39°C, 5% CO2. THP-1(ATCC TIB-202), MT4 (NIH AIDS Reagent Program 120) and MDM (this study) cells were maintained in RPMI-1640 (Invitrogen) supplemented with 10% heat-inactivated FBS, penicillin and streptomycin at 37°C, 5% CO2.

The sex of HEK293T, HEK293, 293A, TZM-bl, HeLa and HeLa-CD4-CCR5 cells are female, while THP-1, MT4, BHK-21 and MDM cells are male. The sex of Rat2 and DF1 are unspecified.

The Rat2 cells expressing myc-tagged TMEM106A orthologues in a doxycycline-inducible manner were generated by transducing Rat2 cells with VSV-G pseudotypted pEasiLV-TMEM106A lentivectors. The transduced cells were treated with doxycycline (Sigma-Aldrich) for 48 h, and E2-Crimson positive cells were collected by fluorescence-activated cell sorting (FACS).

To generate THP-1 cells expressing myc-tagged TMEM106A and NTM-LZ in a doxycycline-inducible manner, the cells were transduced with VSV-G pseudotypted pTripZ-TMEM106A-3myc or pTripZ-NTM-LZ-3myc lentivectors followed by selection in the culture medium containing puromycin (Ameresco).

293T cells expressing myc-tagged TMEM106A in a doxycycline-inducible manner were generated by transducing the cells with VSV-G pseudotypted pTripZ-TMEM106A-3myc lentivectors followed by selection in the culture medium containing puromycin (Ameresco).

To knockout *TMEM106A* in MT4 cells, the cells were transduced with VSV_G pseudotypted lentivectors expressing a control sgRNA or a mix of two sgRNAs, and the transduced cells were selected in culture medium containing puromycin (Ameresco). The knockout efficiency of *TMEM106A* was confirmed by genotyping PCR and qRT-PCR using the follow primers:

Genotype-F: 5′-GCCAGTCTTGAGAACTTCAAACC-3′.

Genotype-R: 5′-CAGGGGCAATGGCTTGTGA-3′.

qKOTM6A-F: 5′-GTGCCTTGTGAAGGAACTGCTG-3′.

qKOTM6A-R: 5′-CCATAGGGAATGAGAGCCACCA-3′.

### Method details

#### Plasmid construction

The coding sequences of TMEM106A, its paralogues and orthologues were either PCR-amplified from a cDNA library or synthesized with codon optimization, and cloned into the expression vector pcDNA4/TO/myc-HisB (Invitrogen) with a triple myc-tag at the C-terminus. Some of them were also cloned into the lentivector pEasiLV-MCS (Goujon et al., 2013) to express proteins in a doxycycline-inducible manner. Unless otherwise indicated, TMEM106A refers to human TMEM106A in this report. The coding sequences include: human TMEM106A (Genbank: NM_001291586.2), human TMEM106B (Genbank: NM_018374.4), human TMEM106C (Genbank: NM_001143842.2), mouse TMEM106A (Genbank: NM_001359325.1), chicken TMEM106B (Genbank: NM_001012558.1), chicken TMEM106C (Genbank: XM_015272611.2), Anole lizard TMEM106A (Genbank: XM_008113178.2), Xenopus tropicalis TMEM106B (Genbank: NM_001016812.2), Xenopus tropicalis TMEM106C (Genbank: NM_001016848.2), zebrafish TMEM106A (Genbank: NM_001128676.1), lamprey TMEM106C (Ensembl version: ENSPMAG00000006874), lamprey TMEM106Bb (Ensembl version: ENSPMAG00000005563.1), *Ciona intestinalis* TMEM106B (Genbank: XM_002123161.5), *Ciona savignyi* TMEM106B (Ensembl version: ENSCSAVG00000006959); lancelet TMEM106Bb (Gene ID: Bb_131540R, http://genome.bucm.edu.cn/lancelet/), hydra TMEM106B (Genbank: XM_002157867.3), and sea anemone TMEM106B (Genbank: XM_001640949.2).

To generate constructs expressing TMEM106A truncation mutants, the coding sequences were PCR-amplified and cloned into pcDNA4/TO/myc-HisB with a triple myc-tag or double Flag-tag at the C-terminus. To generate constructs expressing NTM-linker (NTML) or NTM-leucine-zipper (NTM-LZ), the N-terminal domain and transmembrane domain of TMEM106A were fused with a linker for NTML or with a linker and the GCN4 leucine zipper (O'Shea et al., 1991) for NTM-LZ, with a triple myc-tag at the C-terminus. TMEM106A and NTM-LZ were cloned into pcDNA4/TO/myc-HisB, or the lentivector pTripZ (Horizon) to express TMEM106A and NTM-LZ in a doxycycline-inducible manner. To generate constructs expressing HIV-1 Envelope proteins, the coding sequences of gp160 and gp120 were PCR-amplified and cloned into the expression vector pEF1α (Wang et al., 2019) with a double Flag-tag at the C-terminus. To express gp41 on the cell surface, the coding sequences of the signal peptide, furin site and gp41 were fused in frame, with a double Flag-tag at the C-terminus. pcDNA4-IRES-mCherry expresses mCherry under the translation control of IRES. To construct pcDNA4-IRES-mCherry, IRES and mCherry coding sequence were PCR-amplified, overlapped and cloned into pcDNA4/TO/myc-HisB. gp120 and gp160 coding sequences were PCR-amplified and cloned into pcDNA4-IRES-mCherry to generate pcDNA4-gp120-IRES-mCherry and pcDNA4-gp160-IRES-mCherry, respectively.

To generate the construct expressing myc-tagged lipid raft marker CD230, the coding sequence were PCR-amplified and cloned into pcDNA4/TO/myc-HisB with a triple myc-tag at the C-terminus. To generate the construct expressing fusion protein TMEM106A-mCherry, the coding sequences of TMEM106A and mCherry were PCR-amplified, overlapped and cloned into pcDNA4/TO/myc-HisB. To generate constructs expressing fusion protein TMEM106A-EGFP or gp160-EGFP, the coding sequences were PCR-amplified and cloned into pEGFP-N1 (HonorGene) with an EGFP-tag at the C-terminus.

The shRNA targeting the 3′UTR of human TMEM106A (TM6Ai) and a control shRNA (Ctrli) were prepared by annealing pairs of oligonucleotides and cloning into pSuper-Retro-Puro (OligoEngine). The coding sequence of TMEM106A without the 3′UTR was cloned into pcDNA4/TO/myc-HisB to serve as a rescue TMEM106A-expressing plasmid. The knockdown efficiency was confirmed by RT-qPCR using GAPDH mRNA as an internal control. The targeting sequences of the shRNAs and the primer sequences for RT-qPCR are listed below:

TM6Ai: 5′-TGTGTGTATGAAGTTAACT-3′;

Ctrli: 5′-GCGCGCTTTGTAGGATTCGTT-3′.

qTM6A-F: 5′-CTGCCACCCATCTGATAGTAAG-3′;

qTM6A-R: 5′-GGAACCAGGGACAGTCAATAA-3′;

qGAPDH-F: 5′-TCGGAGTCAACGGATTTG-3′

qGAPDH-R: 5′-GCATCGCCCCACTTGATT-3′.

The CRISPR single-guide RNAs (sgRNAs) targeting the genome of human TMEM106A (*TM6A*-sgRNA) and a control sgRNA (*Ctrl*-sgRNA) were prepared by annealing pairs of oligonucleotides and cloning into pLentiCRISPR v.2 (Addgene 52,961). The targeting sequences of the sgRNAs are listed below:

*TM6A*-sgRNA1: 5′-GGTAAGACGTTTTCCCAGCT-3′;

*TM6A*-sgRNA2: 5′-GGTGGCTCTCATTCCCTATG-3′;

*Ctrl*-sgRNA: 5′-AAATGTGAGATCAGAGTAAT-3′.

#### Virus preparation, infection and detection

To produce VSV-G pseudotyped HIV-1 vector NL4-3luc, pNL4-3-luc and pVSV-G were transfected into 293T cells using Xpregen Transfection Reagent following the manufacturer’|'s instructions (Beijing Yu-Feng Biotechnology) (Connor et al., 1995). The proviral DNA pNLenv-luc was transfected into 293T cells to produce the replication-competent HIV-1virus NLenv-luc (Dang et al., 2006). The titer of NLenv-luc was assayed on HeLa-CD4-CCR5 cells, with luciferase activity in the recipient cells serving as an indicator of the relative viral titer. The titers of NL4-3-R3A (Zhang et al., 2011) and NL4-3 viruses were assayed on TZM-bl cells.

VSV-G pseudotyped MLV-luc vector was produced by transfecting pVSV-G, pHIT60 and pMLV-luc into HEK293 cells (Gao et al., 2002). The replication-competent MLV was produced by transfecting the proviral DNA pNCA into 293T cells (Colicelli and Goff, 1988). For replication-competent MLV, culture supernatants were used to infect Rat2 cells, and viral replication was monitored by measuring RT activity in the culture supernatant. Unless otherwise indicated, in all the transfections of constructs expressing firefly luciferase, a plasmid expressing renilla luciferase was included to serve as a control for transfection efficiency and sample handling.

To assay HIV-1 replication in MT4 cells, 1×10^6^ control or *TMEM106A* knockout cells were infected with NL4-3 virus (0.01 ng p24) for 2 h. The cells were washed twice with PBS and cultured in fresh medium. One half of the cells and medium were transferred to new culture dishes containing fresh medium every day and the remaining culture supernatant was collected to infect TZM-bl indicator cells to monitor viral replication. To assay HIV-1 replication in THP1 cells which express TMEM106A or NTM-LZ in a doxycycline-inducible manner, 2×10^6^ cells were infected with the NL4-3-R3A virus (6 ng p24). The cells were washed once with PBS, split equally into two dishes, and mock treated or treated with doxycycline. One half of the cells and medium were transferred to new dishes containing fresh medium with or without doxycycline every other day and the remaining culture supernatants were collected to infect TZM-bl indicator cells to monitor viral replication.

To assay HIV-1 replication in monocyte-derived-macrophages (MDMs), human monocytes were isolated from PBMC with CD14 Microbeads (Miltenyi Biotec, MACS), cultured in 24-well plates (5×10^5^ per well) and treated with 20 ng/mL hM-CSF (Sangon Biotech) to induce differentiation. On days 7 and 10 of differentiation, the MDMs were transfected twice with a control siRNA or an siRNA targeting human TMEM106A (GenePharma) using Lipofectamine 2000 (Thermo Fisher) following the manufacturer's instructions. At 4 h posttransfection, the MDMs were infected with the NL4-3-R3A virus (10 ng p24). Aliquots of the culture supernatants were collected every other day to infect TZM-bl indicator cells to monitor viral replication. The knockdown efficiency of the siRNAs was confirmed by RT-qPCR using β-actin mRNA as an internal control. The target sequences of the siRNAs and the primer sequences are listed below.

Ctrli: 5′-UUCUCCGAACGUGUCACGUTT-3′;

TM6Ai: 5′-GGGAGAAGUGGUGACAGAATT-3′.

qTM6A-F: 5′-CTGCCACCCATCTGATAGTAAG-3′;

qTM6A-R: 5′-GGAACCAGGGACAGTCAATAA-3′;

qACTIN-F: 5′-CACCATTGGCAATGAGCGGTTC-3′;

qACTIN-R: 5′-AGGTCTTTGCGGATGTCCACGT-3′.

The production and titration of replication-competent Sindbis virus SINV-nLuc (Wang et al., 2016), vesicular stomatitis virus VSV-GFP (Dalton and Rose, 2001) and influenza A virus IAV-Gluc (Pan et al., 2013) have been previously described. To assay the antiviral activity of endogenous cTMEM106B, DF-1 cells were transfected with a control siRNA or siRNAs targeting cTMEM106B (GenePharma) using Lipofectamine 2000 (Thermo Fisher) following the manufacture's instructions. At 24 h posttransfection, the cells were infected with SINV-nLuc at an MOI of 0.0005 at 39°C, VSV-GFP at an MOI of 0.0001 at 37°C, or IAV-GLuc at an MOI of 0.0001 at 39°C for 1 h. The cells were washed twice with PBS and cultured in DMEM supplemented with 2% fetal bovine serum. At various time points, the cells infected with SINV-nLuc were lysed and luciferase activity was measured. To monitor the propagation of VSV-GFP virus, the culture supernatants were collected at various time points and stored at −80°C. Virus samples were titrated in triplicate on BHK-21 cells. The cells were inoculated with 10-fold serial dilutions of the virus (diluted in serum-free DMEM medium). At 1 h postinfection, the inoculum was removed and cells were covered with DMEM overlay containing 1% methylcellulose (Sigma-Aldrich) and 2% FBS. At 18–24h postinfection, overlay was removed and the cells were stained with 0.2% crystal violet in 20% ethanol, followed by plaques enumeration. To assay IAV-Gluc replication, aliquots of the culture supernatants were taken to measure Gaussia luciferase activity. The knockdown efficiency of the siRNAs was confirmed by qRT-PCR using GAPDH mRNA as an internal control. The targeting sequences of the siRNAs and primer sequences for RT-qPCR are listed below.

cCtrli: 5′-UUCUCCGAACGUGUCACGUTT-3′;

cTM6Bi-1: 5′-CCAGGGAACGGGAAGAAUUTT-3′;

cTM6Bi-2: 5′-GCAUGUAAUGCAUAGGAAATT-3′.

qcTM6B-F: 5′-TAGTGTGGAATGACTGCTCTTG-3′;

qcTM6B-R: 5′-AGGCCACCATGTTTCCTATG-3′;

qcGAPDH-F: 5′-CAG AACATCATCCCAGCGTC-3′;

qcGAPDH-R: 5′-CAGGTCAGGTCAACAACAGAG-3′.

To assay the antiviral activity of TMEM106A orthologues against MLV replication, Rat2 cells expressing myc-tagged TMEM106 proteins in a doxycycline-inducible manner were seeded in duplicate in six-well plates (3×10^5^ cells per well) and infected with MLV. At 3 h postinfection, cells were washed once with PBS and cultured in DMEM supplemented with 2% fetal bovine serum with or without 1 μg/mL doxycycline. One-half of the culture supernatant was replaced with fresh medium with or without doxycycline every day. The culture supernatants were assayed for reverse transcriptase activity to monitor the viral replication. For the reverse transcriptase assay, 10 μL of the supernatant was incubated with 50 μL reaction buffer [50 mM Tris–HCl (pH 8.0), 150 mM KCl, 5 mM DTT, 5 mM MgCl_2_, 0.1% Triton X-100, and 0.5 mM EGTA, 5 mg/ml oligo dT, 10 mg/mL poly(rA), 3 μCi of ^32^P-TTP] at room temperature for 1 h and then 4 μL of the reaction was dropped on DE81 paper (Whatman). The paper was washed three times with 2 × SSC buffer, dried, and exposed to Phophoimager or X-ray films.

For Western analysis of virion particles, culture supernatants were loaded on a 25% sucrose cushion in TNE buffer (50 mM Tris-Hcl, pH 7.4; 100 mM NaCl; 1 mM EDTA) and centrifuged at 25,000 rpm for 2 h at 4°C. The pelleted virions were either resuspended in SDS-PAGE loading buffer or applied to 10–50% linear sucrose gradient centrifugation at 25,000 rpm for 16 h at 4°C. Fractions were collected, and proteins were precipitated by Trichloroacetic acid/actone and analyzed by Western blotting. The methods of digesting virion particles with subtilisin have been described previously (Prasad et al., 2009). Briefly, virus-containing culture supernatants were centrifuged through 25% sucrose cushion at 25,000 rpm for 2 h at 4°C. The pelleted virions were resuspended in TNE buffer and split equally, mock treated or treated with subtilisin at 37°C for 4 h. After digestion, PMSF was added to inhibit the protease and the digested virions were pelleted through 25% sucrose cushion at 31,600 rpm for 90 min at 4°C, followed by Western analysis.

#### Co-immunoprecipitation assay

293T cells in 60 mm dishes were transfected with 2 μg total plasmids. At 48 h posttransfection, cells were lysed in CelLyticM^TM^ M Cell Lysis Reagent (Sigma-Aldrich) or Co-IP buffer (30 mM HEPES, pH 7.5; 150 mM NaCl; 0.5% NP-40; 30 mM EDTA) supplemented with protease inhibitor cocktail (Roche) for 15 min on ice. The lysate was clarified by centrifugation for 15 min at 12,000 rpm at 4°C. The clarified cell lysates were mixed with Anti-c-Myc Agarose Affinity Gel (Sigma-Aldrich) or the Anti-Flag M2 Affinity Gel (Sigma-Aldrich) at 4°C for 3 h. The beads were washed three times with TBST (20 mM Tris-HCl pH7.6, 150 mM NaCl, 0.1% Tween-20) and the bound proteins were resolved on SDS-PAGE electrophoresis, transferred to PVDF membrane and detected by Western blotting.

#### Membrane flotation assay

Cell fractionation and equilibrium sucrose density gradient centrifugation assays were modified from those reported previously (Pietilä et al., 2018). Briefly, 5×10^6^ 293T cells were trypsinized, washed with PBS twice and resuspended in 0.5 mL TE buffer (10 mM Tris-HCl pH7.5, 4 mM EDTA) supplemented with the complete protease inhibitor cocktail (Roche). The samples were prepared using a Dounce homogenizer to remove unlysed cells and nuclei as described previously (Pietilä et al., 2017). After low-speed centrifugation (510 g, 10 min, 4°C), the postnuclear supernatant (PNS) was adjusted to 150 mM NaCl and mixed with 85% (wt/vol) sucrose in TNE buffer (25 mM Tris-HCl pH7.5, 150 mM NaCl, 4 mM EDTA) and placed at the bottom of a centrifuge tube. On top of this PNS-containing 73% (wt/vol) sucrose mixture was layered 65% (wt/vol) sucrose in TNE and 10% (wt/vol) sucrose in TNE buffer. The gradients were centrifuged at 100,000 g for 18 h at 4°C. Eleven fractions in all, 1 ml each, were collected for Western blotting analysis.

#### Confocal microscopy

To show the interaction of TMEM106A with lipid raft, a plasmid expressing myc-tagged lipid raft marker CD230 was cotransfected into 293T cells with a plasmid expressing TMEM106A-EGFP. At 16 h posttransfection, cells were fixed for 1 h with 4% paraformaldehyde, washed with PBS, and permeabilized with 0.2% Triton X-100. The cells were stained with anti-myc antibody, TRITC-conjugated secondary antibody and DAPI (Beyotime biotechnology), washed 3 times with PBS, and photographed using a Laser Confocal Microscope (Zeiss LSM700). To show the interaction of TMEM106A with gp160, a plasmid expressing TMEM106A-mCherry was cotransfected into 293T cells with a plasmid expressing gp160-EGFP. To show the interaction of TMEM106A with Gag, a plasmid expressing myc-tagged TMEM106A was cotransfected into 293T cells with pHIV-Gag-iGFP-ΔEnv, which expresses a Gag-GFP fusion protein (Hubner et al., 2007; Micsenyi et al., 2013). At 16 h posttransfection, cells were fixed for 1 h with 4% paraformaldehyde, washed with PBS 3 times, and permeabilized with 0.2% Triton X-100. Myc-tagged TMEM106A was stained with anti-myc antibody, TRITC-conjugated secondary antibody and DAPI, washed 3 times with PBS, and photographed using a Laser Confocal Microscope (Zeiss LSM700).

#### Transmission electron microscopy

293T cells were transfected with pNL4-3luc together with an empty vector or a plasmid expressing TMEM106A. At 36 h posttransfection, cells were washed once with PBS and fixed with 2.5% glutaraldehyde (Sigma-Aldrich) for 10 min at room temperature. The cells were scraped and transferred to Eppendorf tubes and centrifuged for 3 min at 3,000 rpm. The cell pellets were washed 3 times with PB buffer (Phosphate Buffer, pH7.2) and fixed with 1% OsO4 for 2 h at room temperature. The samples were washed three times with PB buffer, dehydrated through a series of ethanol (30%, 50%, 70%, 90%, 95%, 100%, 100%) for 10 min each, and infiltrated with and embedded in epon. After polymerizing, ultrathin sections (∼70 nm) were cut and double stained with 2% uranylacetate and 0.3% lead citrate and examined with a transmission electron microscope at an acceleration voltage of 120 kV (Tecnai Spirit; FEI).

### Quantification and statistical analysis

The Excel software (Microsoft) and GraphPad Prism were used to determine average values and standard deviations. Mean values ±SD were calculated from three independent experiments unless otherwise indicated, and p values were calculated using the two-tailed paired Student's *t* test. ∗ denotes p< 0.05; ∗∗ denotes p< 0.01;∗∗∗ denotes p< 0.01; n.s. denotes p> 0.05.

## Data Availability

• This study did not generate any original code.
