# Supplementary material for: TMEM106A inhibits enveloped virus release from cell surface
Source: iScience. 2022 Feb 1;25(2):103843. doi: 10.1016/j.isci.2022.103843 (PMC8844723; doi:10.1016/j.isci.2022.103843)
Supplement: Document S1. Figures S1–S14 and Table S1 [file mmc1.pdf]

iScience, Volume *25*

Supplemental information

TMEM106A inhibits enveloped virus release from cell surface

Dexin Mao, Feixiang Yan, Xiaolin Zhang, and Guangxia Gao

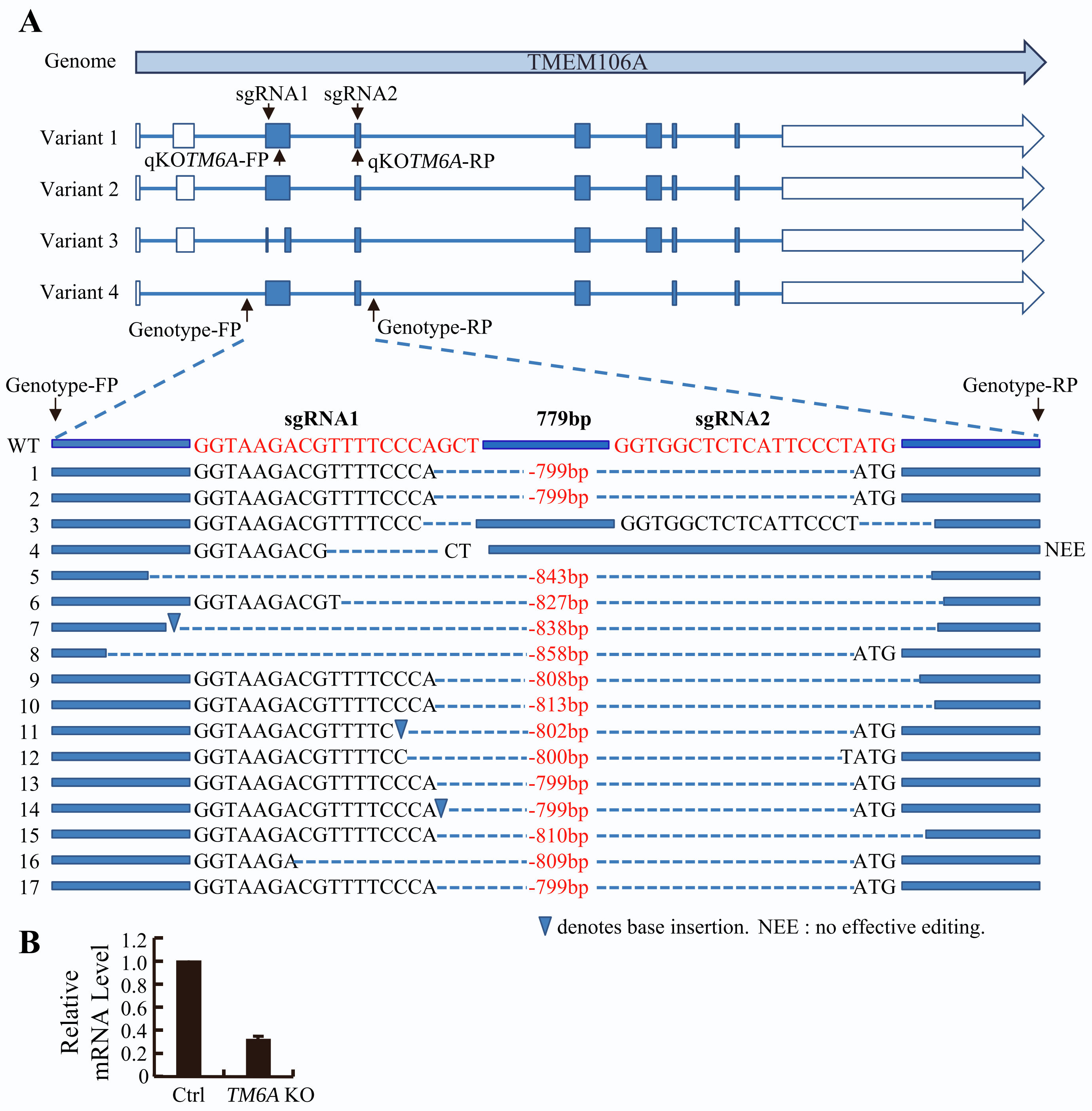

Figure S1. Validation of the knockout of *TMEM106A*. Related to Figure 1D. (A) Upper, schematic representation of *TMEM106A* genomic DNA, sgRNA target sites and PCR primers. There are four putative splicing isoforms (Variant 1-4). Shaded and open boxes represent exons of ORFs and UTR, respectively. Lines represent introns. Arrows indicate the sgRNA target and PCR primer sites. qKO*TM6A*-FP: forward primer for RT-qPCR; qKO*TM6A*-RP: reverse primer for RT-qPCR; Genotype-FP: forward primer for PCR genotyping; Genotype-RP: reverse primer for PCR genotyping. Lower, genotyping of pooled *TMEM106A* knockout MT4 cells was performed by PCR-amplifying target genomic DNA and sequencing of randomly picked cloned PCR products. Dashed lines indicate deleted bases. **(B)** *TMEM106A* knockout efficiency was analyzed by RT-qPCR. The relative mRNA levels of TMEM106A were normalized with those of β-actin. Data presented are means ± SD of three independent experiments.

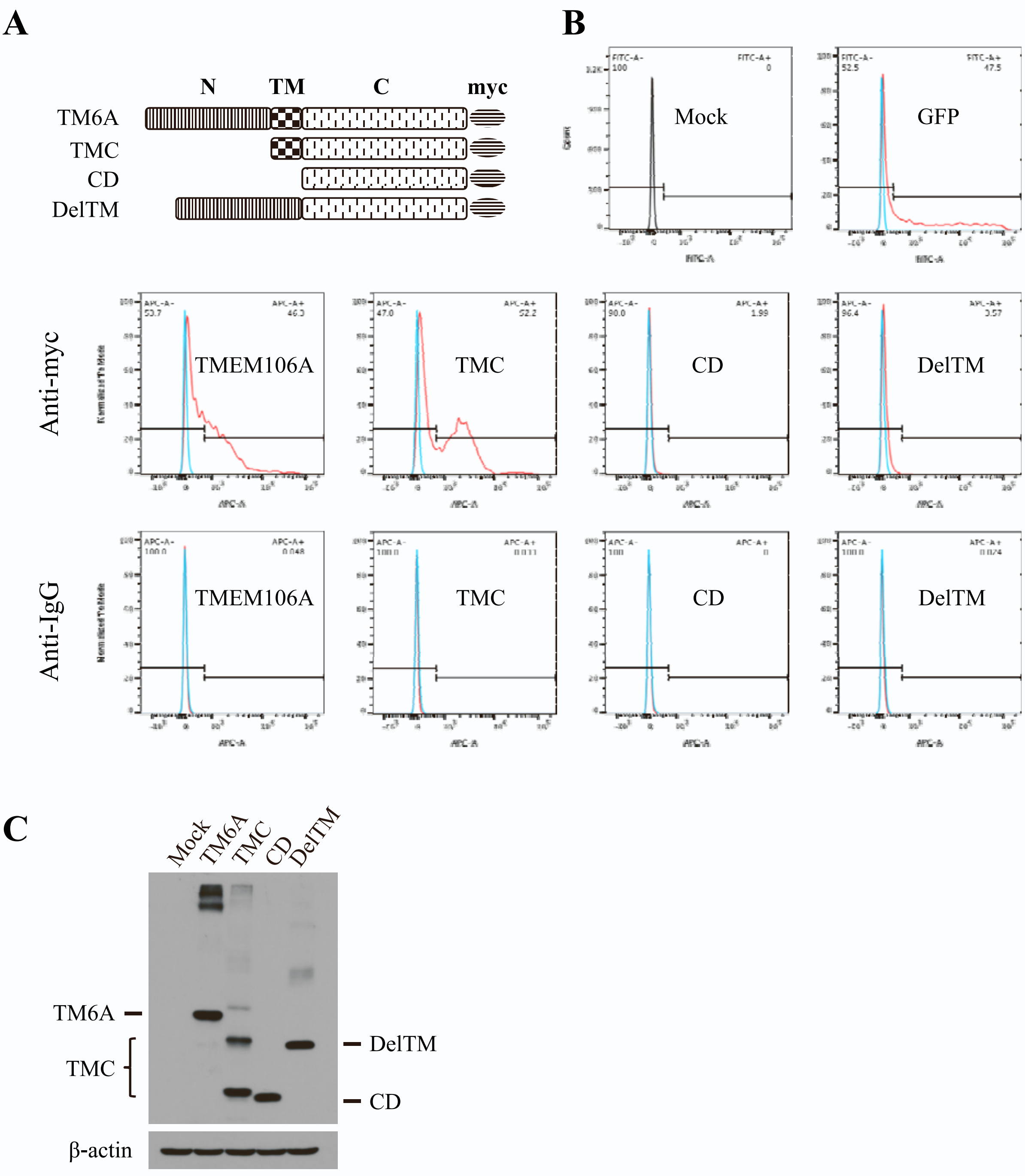

Figure S2. Cell surface localization of TMEM106A truncation mutants. Related to Figure 2B. (A) Schematic representation of TMEM106A truncation mutants. **(B)** and **(C)** 293T cells were transfected with a plasmid expressing a C-terminally myc-tagged TMEM106A truncation mutant. A plasmid expressing GFP was included to serve as a control. At 24 h posttransfection, cells were surface stained with anti-myc or control antibody and APC-conjugated secondary antibody for FACS analysis (B) or lysed for Western analysis (C).

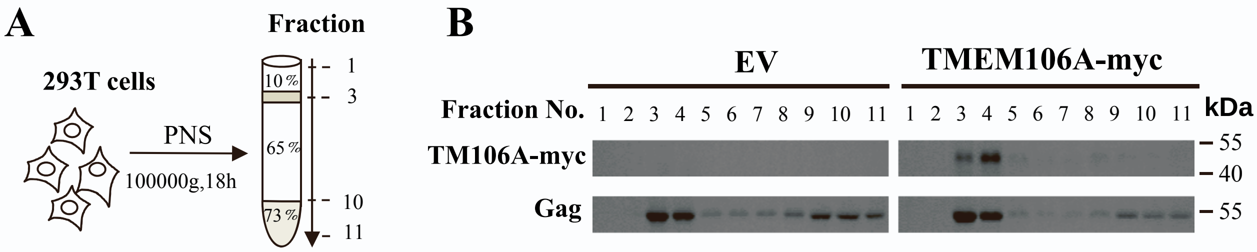

Figure S3. TMEM106A doesn't affect Gag membrane binding. Related to Figure 2. (A) Schematic illustration of the experimental procedure. 293T cells were transfected with pNL4-3luc, with or without a plasmid expressing TMEM106A. At 48 h posttransfection, cells were lysed in hypotonic buffer and the lysates were centrifuged to remove the cell debris and nuclei. The postnuclear supernatant (PNS) was analyzed by the membrane flotation assay. The membrane-associated proteins are expected to be in fractions 3 and 4, and the proteins not associated with membrane are expected to be in other fractions. **(B)** Protein levels in each fraction were analyzed by Western blotting.

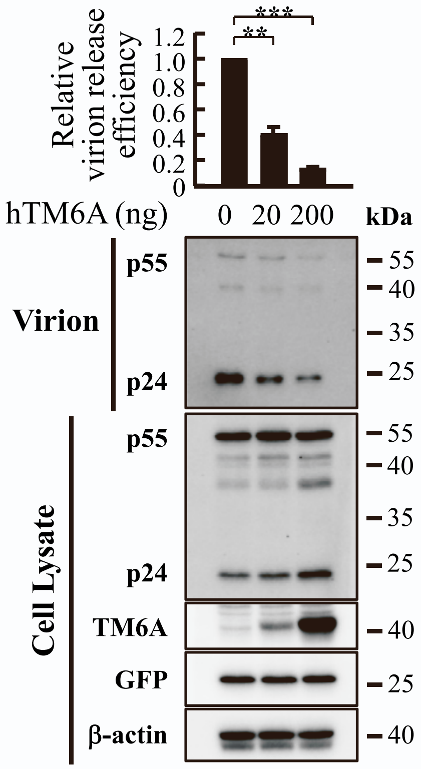

Figure S4. TMEM106A inhibition of HIV-1 virion release increased cell-associated p24 level. Related to Figure 2. 293A cells were transfected with pNL4-3luc and increasing amounts of a plasmid expressing myc-tagged TMEM106A. A plasmid expressing GFP was included to serve as a control for transfection efficiency and sample handling. At 48 h posttransfection, cells were washed once with PBS and lysed directly, without trypsinization. The cell lysates and culture supernatants were subjected to Western Blotting analysis. Virion release efficiency was determined as the virion-associated p24 level divided by the total Gag protein level in the cell lysate. The relative virion release efficiency from the producer cells transfected with the empty vector was set as 1. Data presented are means ± SD of three independent experiments. ** denotes $p<0.01$; *** denotes $p<0.001$

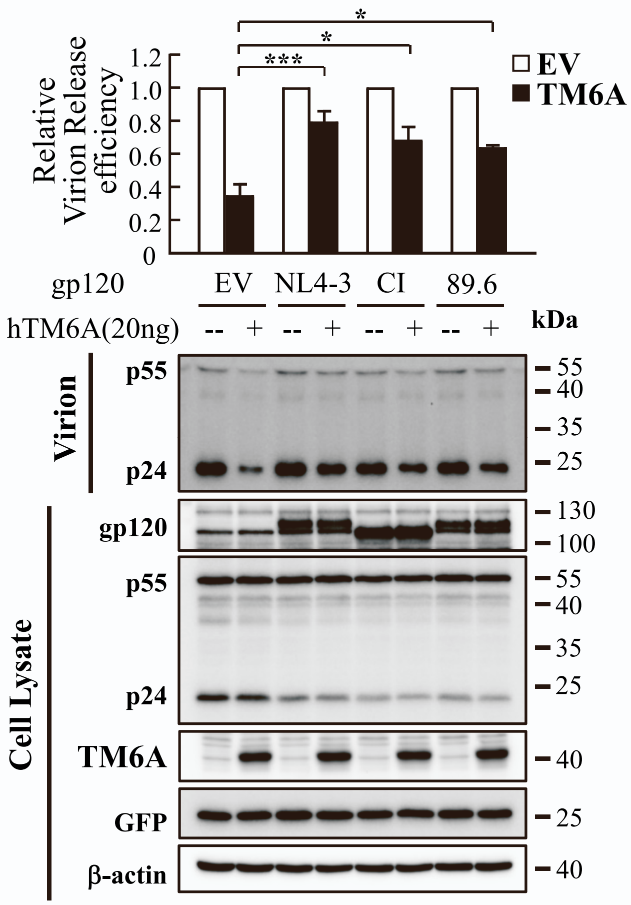

Figure S5. gp120 from different HIV-1strains can antagonize TMEM106A. Related to Figure 3C. 293A cells were transfected with pNL4-3luc together with an empty vector (EV) or a plasmid expressing gp120 from HIV-1 strain NL4-3, CI or 89.6, with or without a plasmid expressing TMEM106A-myc. A plasmid expressing GFP was included to serve as a control for transfection efficiency and sample handling. At 48 h posttransfection, the producer cells and culture supernatants were analyzed by Western blotting. Virion release efficiency was determined as the virion-associated p24 level divided by the total Gag protein level in the cell lysate. The relative virion release efficiency from the producer cells transfected with the empty vector was set as 1. Data presented are means ± SD of three independent experiments. * denotes $p<0.05$; *** denotes $p<0.001$.

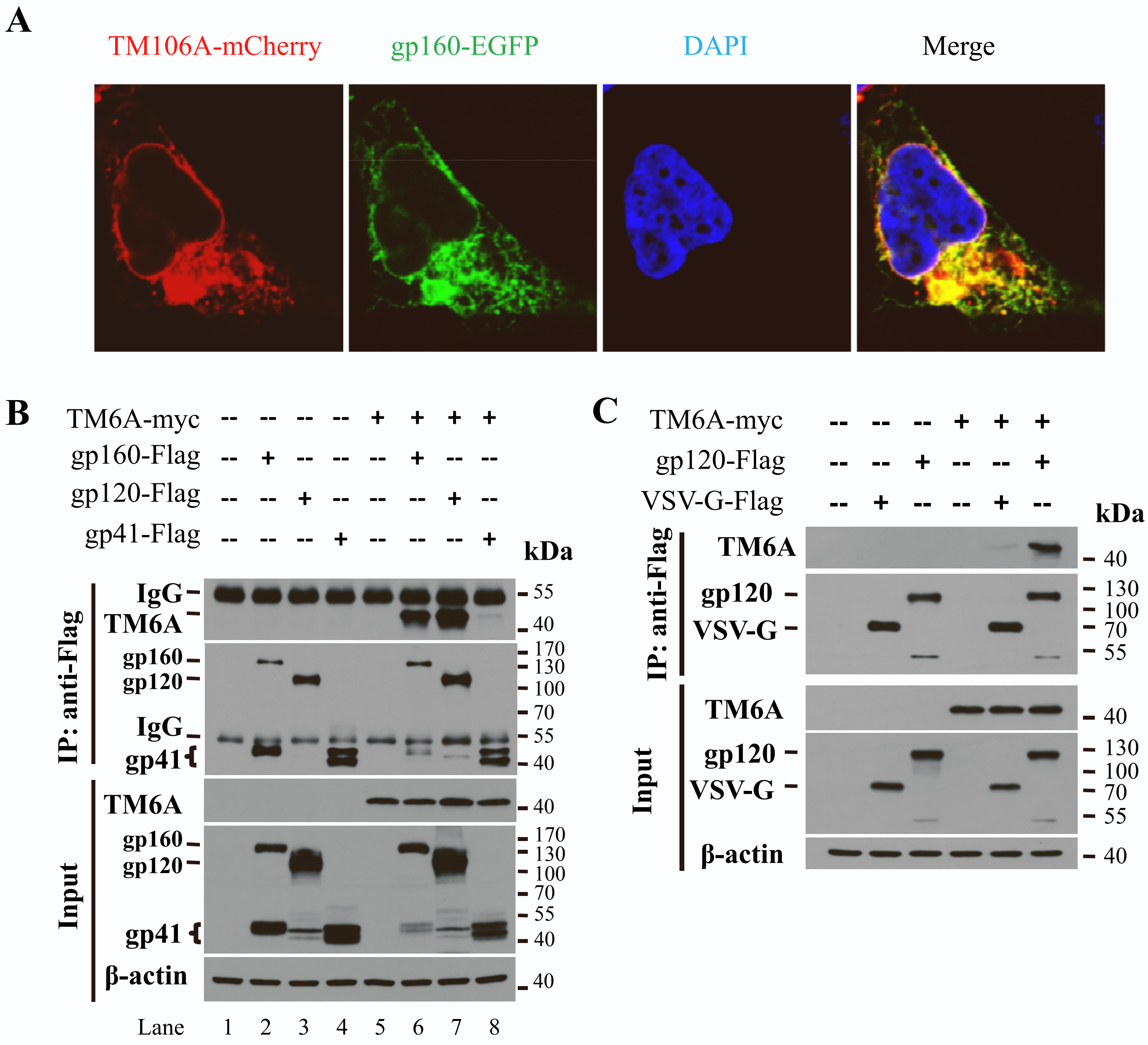

Figure S6. TMEM106A interacts with HIV-1 gp120 but not gp41 or VSV-G. Related to Figure 4. **(A)** 293T cells were co-transfected with plasmids expressing the proteins indicated. At 16 h posttransfection, cells were stained with 4',6-diamidino-2-phenylindole (DAPI) and subjected to confocal microscopy analysis. **(B)** and **(C)**293T cells were transfected with plasmids expressing the proteins indicated. At 48 h posttransfection, cells were lysed and the lysates were immunoprecipitated with the antibody indicated, followed by Western blotting.

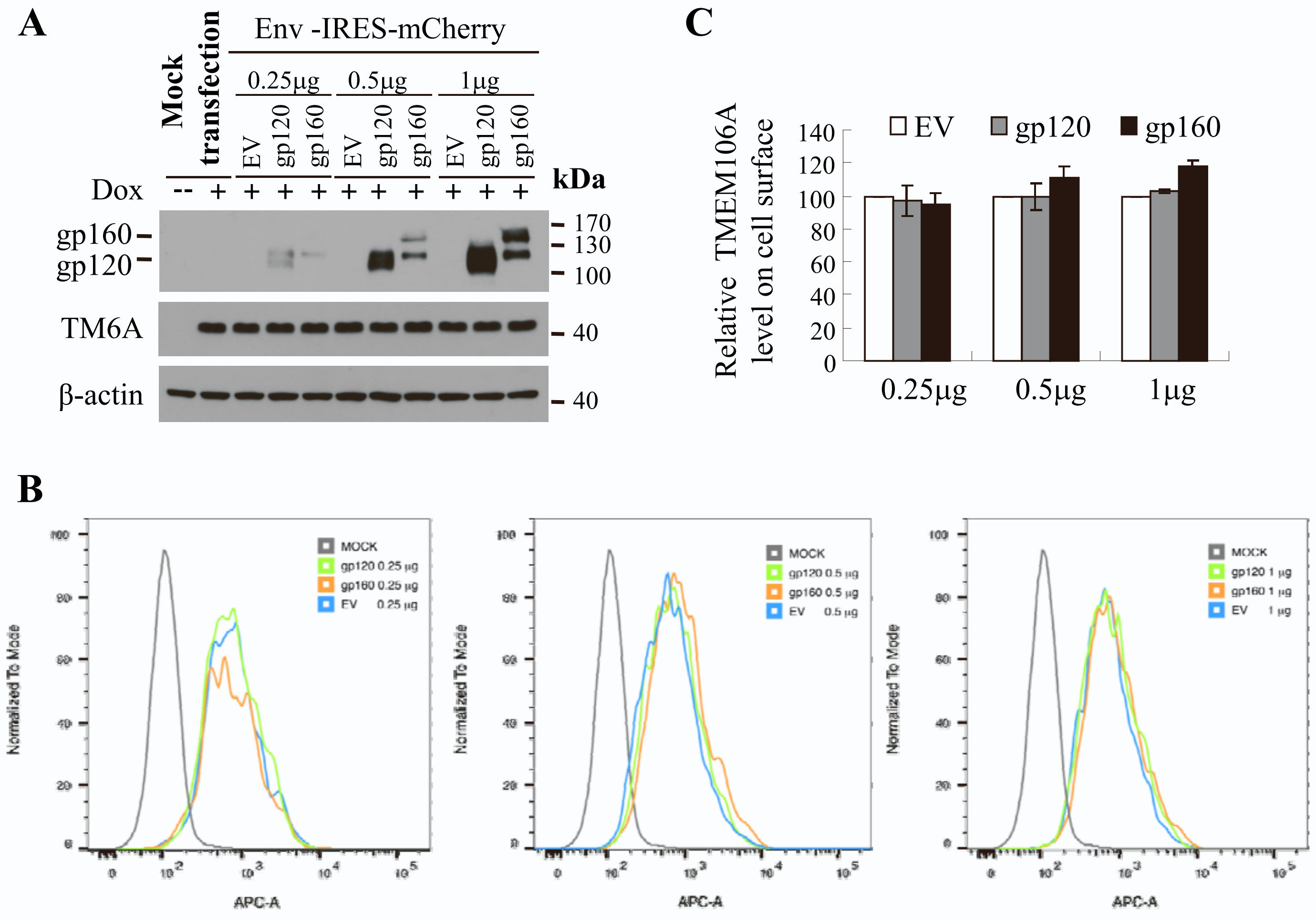

Figure S7. HIV-1 gp120 does not affect TMEM106A levels on cell surface. Related to Figure 4. 293T cells expressing C-terminally myc-tagged TMEM106A in a doxycycline-inducible manner were transfected with an empty vector (EV) or a plasmid expressing gp120 or gp160 and mCherry that was under the translation control of IRES. At 48 h posttransfection, cells were analyzed. **(A)** Western blotting analysis of protein expression. **(B)** and **(C)** Cells were surface-stained with anti-myc antibody and APC-conjugated secondary antibody, followed by FACS analysis. The mCherry positive cells were gated and analyzed. **(B)** Representative flow cytometry profiles. **(C)** Summary of the relative levels of TMEM106A on cell surface, as indicated by the geometric means fluorescence of APC. Data presented are means ± SD of three independent experiments.

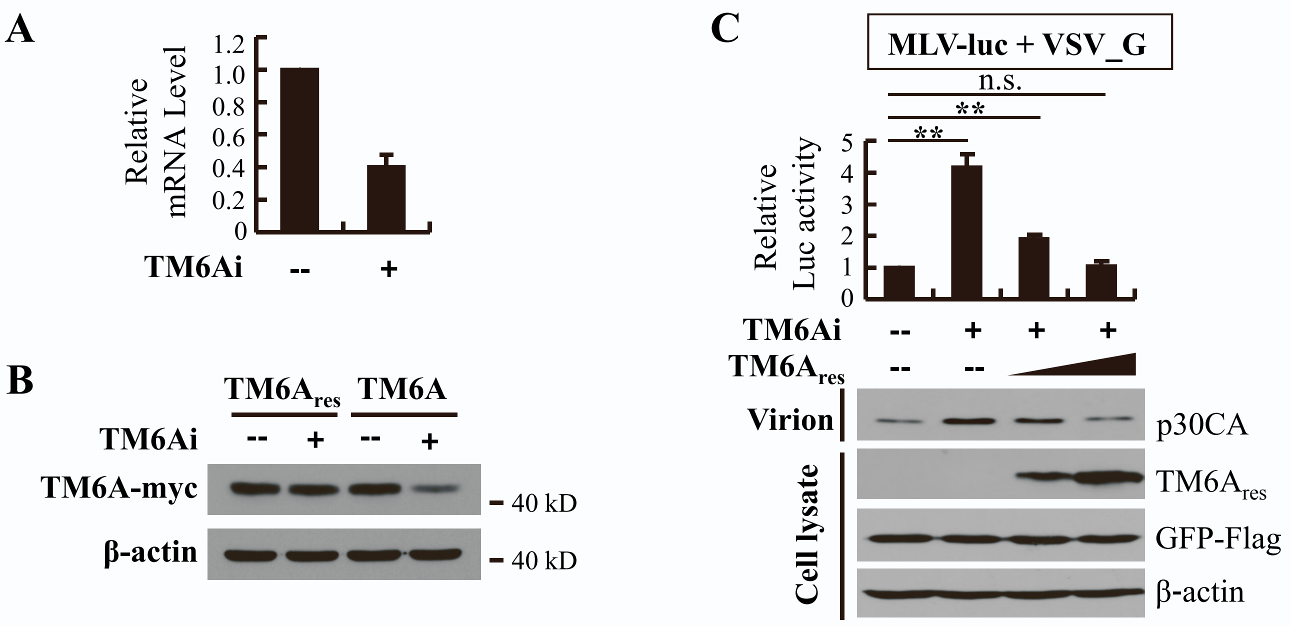

Figure S8. Downregulation of endogenous TMEM106A enhanced MLV production. Related to Figure 6. (A) HEK293 cells were transfected with a plasmid expressing a control shRNA or an shRNA targeting human TMEM106A. At 48 h posttransfection, the mRNA levels of TMEM106A were analyzed by RT-qPCR. The relative mRNA levels of TMEM106A were normalized with those of GAPDH. **(B)** 293T cells were transfected with a plasmid expressing myc-tagged TMEM106A or a rescue plasmid, together with a plasmid expressing a control shRNA or an shRNA targeting human TMEM106A. At 48 h posttransfection, TMEM106A protein levels were measured by Western blotting. **(C)** HEK293 cells were transfected with plasmids producing VSV-G pseudotyped MLV-luc, together with a plasmid expressing the shRNA targeting TMEM106A and increasing amounts of a rescue plasmid expressing TMEM106A-myc. At 48 h posttransfection, the culture supernatants were used to infect HeLa cells. The cell lysates were analyzed for protein expressions, and the supernatants were assayed for protein expression and relative amount of infectious virion particles. The relative luciferase activity in the recipient cells infected with the virus produced in the control cells was set as 1. Data presented are means ± SD of three independent experiments. ** denotes $p<0.01$; n.s. denotes $p>0.05$.

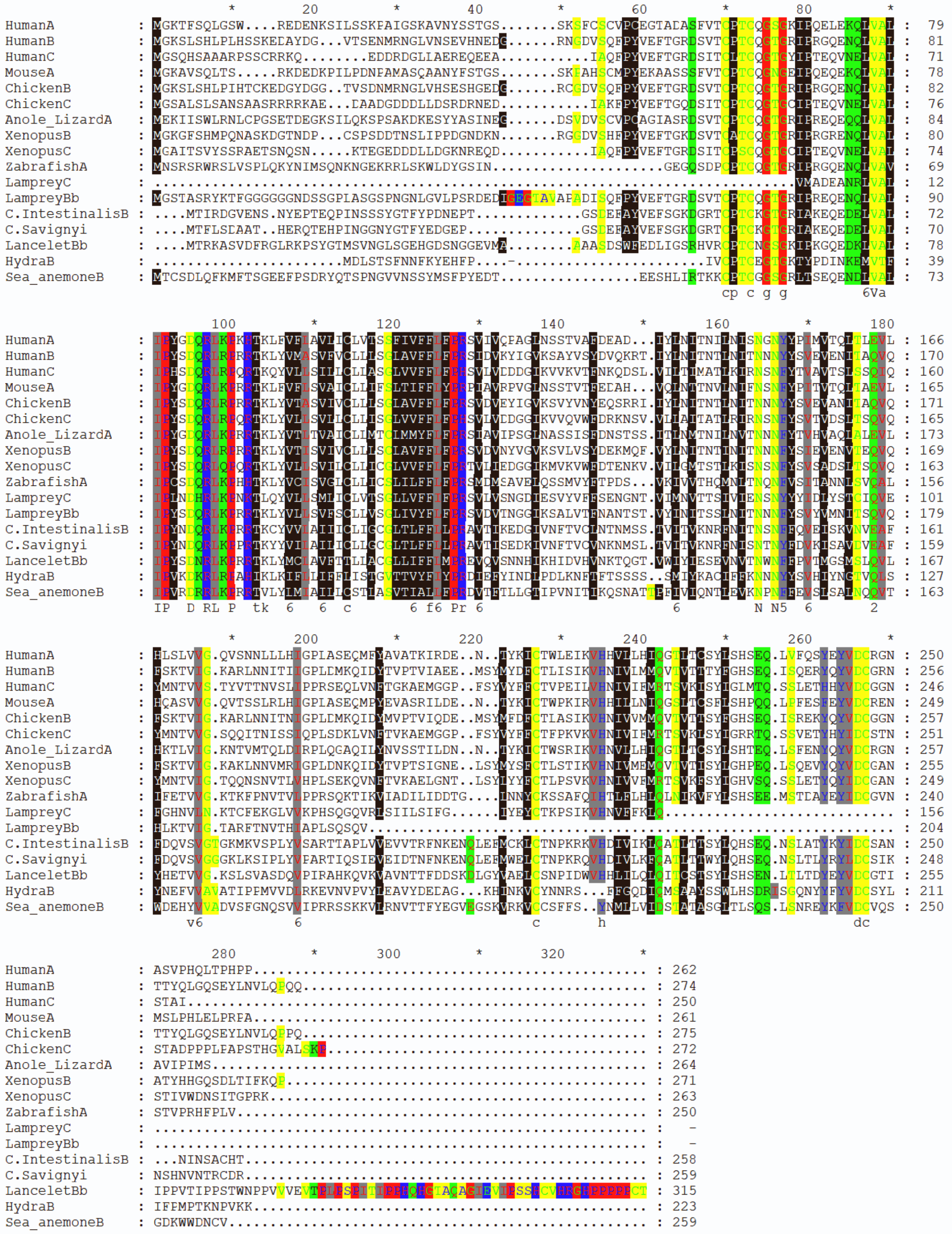

Figure S9. Sequence alignment of TMEM106 proteins. Related to Figure 6. The multiple sequences were aligned by the MEGA7 and colored by GeneDoc.

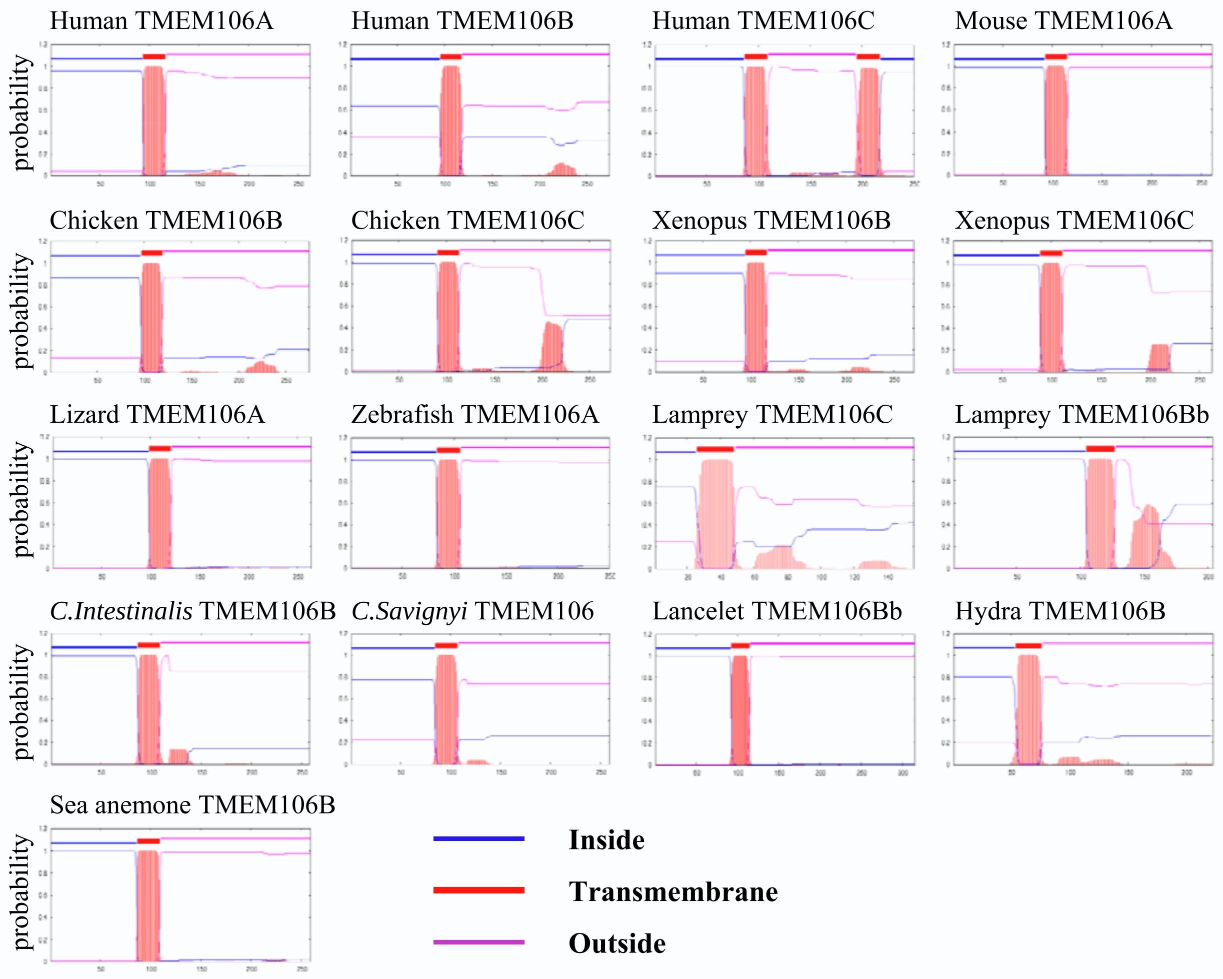

Figure S10. Sequence analysis of the predicted transmembrane domain(s) of TMEM106 proteins. Related to Figure 6.

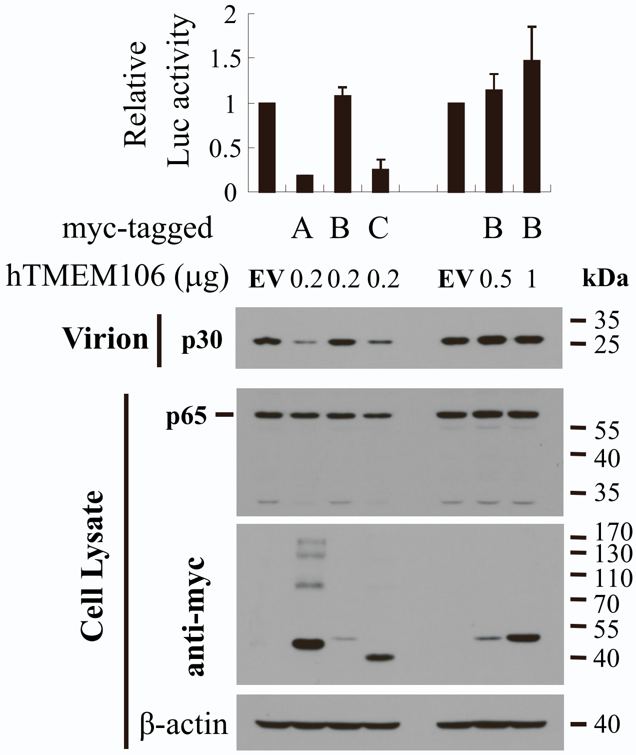

Figure S11. Human TMEM106A and TMEM106C, but not TMEM106B inhibit MLV production. Related to Figure 6. The MLV-luc vector-producing plasmids were transfected into 293T cells with an empty vector (EV) or the indicate amount of a plasmid expressing myc-tagged human TMEM106A, 106B or 106C. At 48 h posttransfection, the producer cells and culture supernatants were analyzed by Western blotting and the culture supernatants were also used to infect recipient cells. At 48 h postinfection, the luciferase activity in the recipient cells was measured. The relative luciferase activity in the recipient cells infected with the virus produced in the control cells was set as 1. Data presented are means ± SD of three independent experiments.

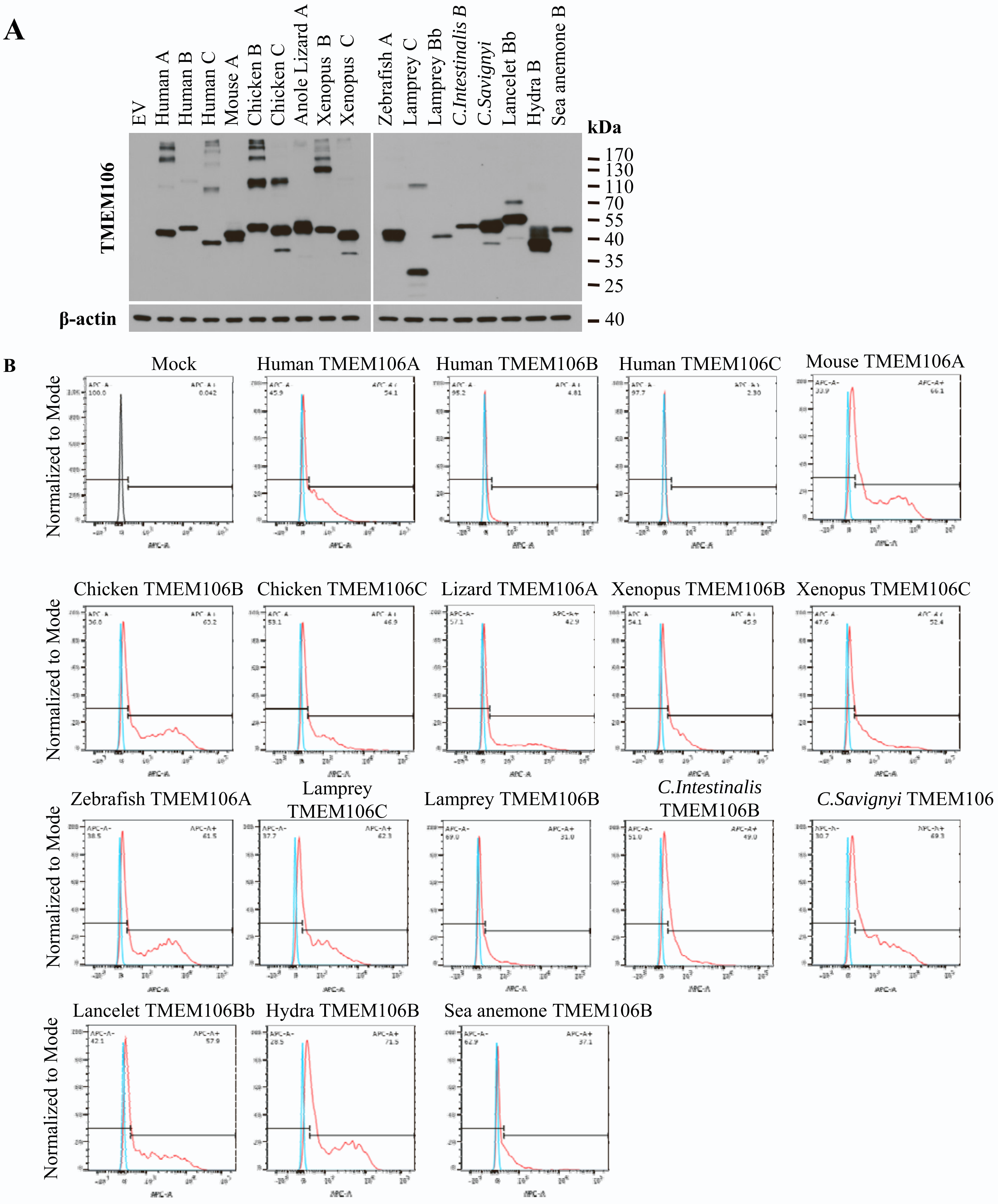

Figure S12. Cell surface localization of TMEM106 proteins. Related to Figure 6. 293T cells were transfected with a plasmid expressing the C-terminally myc-tagged TMEM106 protein. At 24 h posttransfection, the cells were either lysed for Western analysis (A) or surface stained with anti-myc antibody and APC-conjugated secondary antibody for FACS analysis (B).

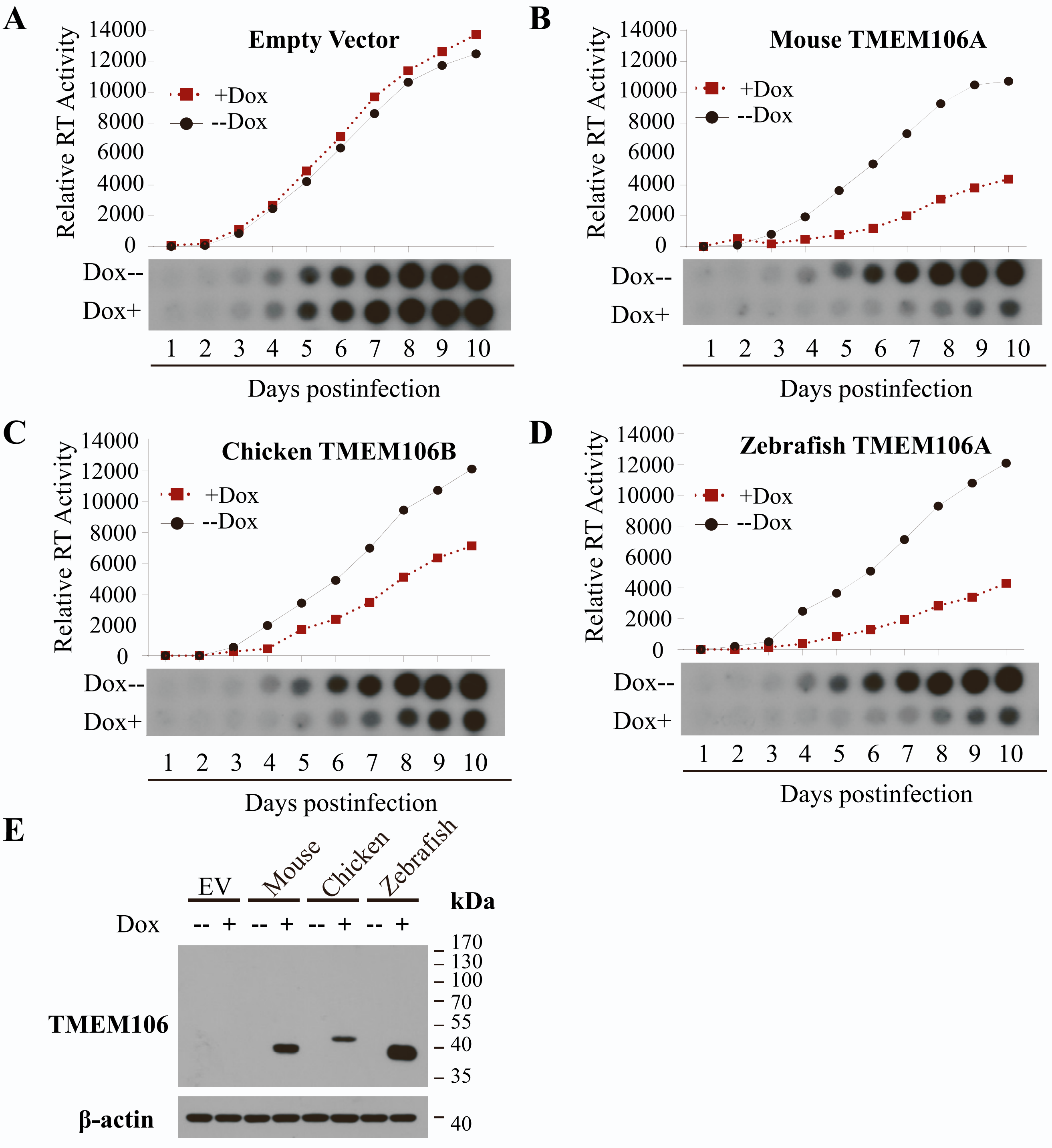

Figure S13. TMEM106A orthologues inhibit MLV replication. Related to Figure 6. Rat2 cells expressing myc-tagged TMEM106 protein in a doxycycline-inducible manner were infected with MLV. **(A-D)** At the time points indicated, culture supernatants were collected and assayed for reverse transcriptase (RT) activity. The relative RT activities quantitated with Phophoimager were used to plot the replication curve. Data presented are representative of two independent experiments. **(E)** Induced expression of the TMEM106 proteins were confirmed by Western blotting at the end of the assays.

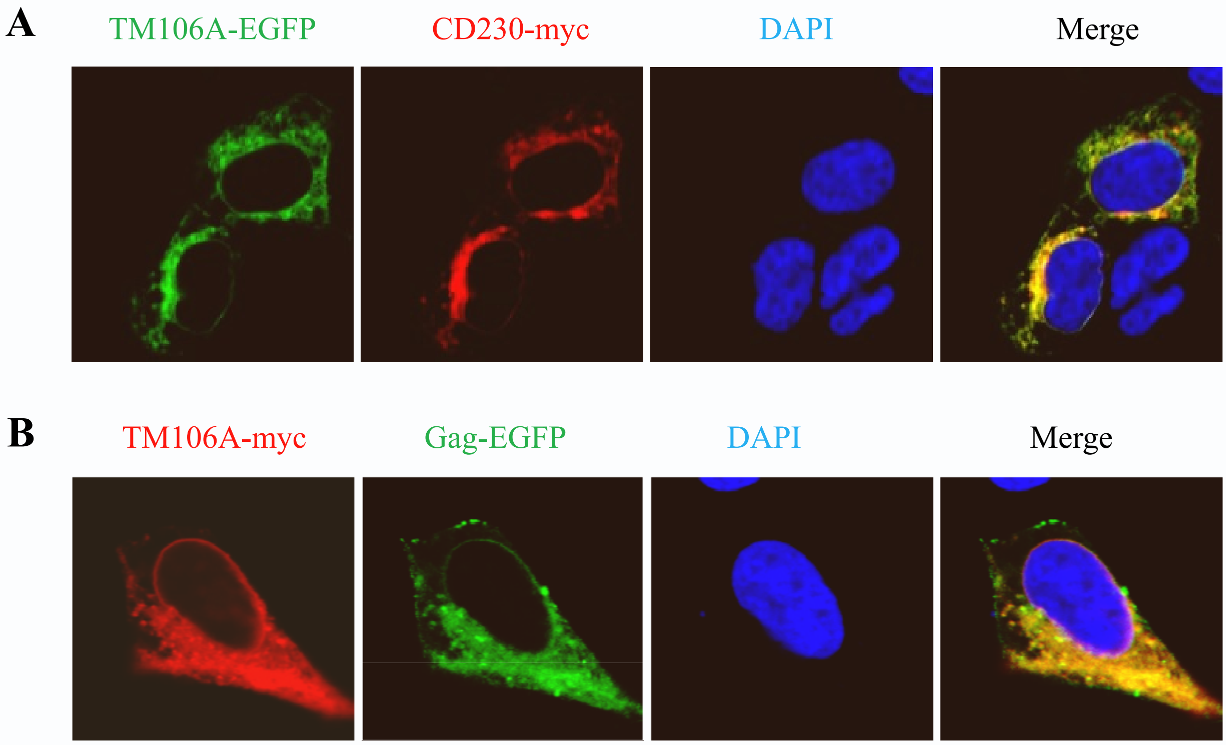

Figure S14. TMEM106A co-localizes with Gag and CD230. Related to Figure 7. 293T cells were co-transfected with plasmids expressing the proteins indicated. At 16 h posttransfection, cells were stained with anti-myc antibody and TRITC-conjugated secondary antibody, and subjected to confocal microscopy analysis.

Table S1. ISGs screened for activity to inhibit the production of VSV-G pseudotyped NL4-3luc. Related to Figure 1.

Gene Name	Fold Inhibition	Gene Name	Fold Inhibition	Gene Name	Fold Inhibition	Gene Name	Fold Inhibition
Empty Vector	1	B3GNT2	1.544	IFI27	0.989	GALM	0.751
SLAMF8	15.403	CASP4	1.513	TMEM45B	0.987	BCL2A1	0.728
TMEM106A	13.490	NEXN	1.463	NAT8L	0.983	GPR84	0.715
SHFL	10.687	ARHGAP25	1.436	EGR1	0.982	USP18	0.710
FTSJD2	8.086	SP140	1.434	LHFPL1	0.972	NCOA7	0.701
GSDMD	6.933	RNF147	1.425	44L	0.922	TIMD2	0.699
SLC38A5	5.855	REC8	1.419	CREM	0.913	RTP4	0.692
MC3R	4.530	MFN1	1.417	CD164	0.910	FAM109B	0.690
MX2	4.263	MSMB	1.416	PARP3	0.909	PDE6H	0.688
TMEM217	4.022	RNF213	1.409	RASGPR3	0.900	WDFY1	0.683
BRWD1	3.222	NFKBIE	1.366	CCDC146	0.894	FCGR2A	0.663
MYO5C	2.641	ECE1	1.354	PLAC8	0.892	APOL6	0.647
PIM1	2.599	MOXD1	1.349	BFSP2	0.877	XAF	0.603
ASPHD2	2.599	ENDOD1	1.319	IFI6	0.872	CFLAR	0.592
NPHP1	2.557	PLEKHG7	1.299	RNASET2	0.856	SELL	0.585
NAPSA	2.543	CKB	1.239	TOR1B	0.848	TMEM229B	0.580
SNX9	2.480	FGD2	1.234	GBP4	0.841	INPP1	0.546
LGAL3BP	2.442	ST3GAL5	1.143	MVP	0.830	TPPP2	0.514
DDAH2	2.302	IER3	1.125	CXORF21	0.812	LMO2	0.479
TIPARP	2.226	TEAD4	1.087	C14ORF149	0.798	IFIT5	0.471
BOC	2.110	CHI3L2	1.083	FNDC5	0.786	IFI44	0.468
PDS5A	1.926	TMEM140	1.076	MS4A12	0.783	ETV4	0.394
PHLDA2	1.925	DDX60	1.073	MR1	0.775	GLYATL1	0.379
AZU1	1.917	XRN2	1.062	CD69	0.764	CASP10	0.348
FRMD3	1.747	TKTL1	1.044	EPSTI1	0.763	SEMA4G	0.335
AGPAT4	1.734	IDH1	1.015	TRIM31	0.760	RNUX3	0.288
ENTPD1	1.661	DCLRE1C	1.006	GSN	0.760	TNFRSF1A	0.246
MGC87042	1.624	C20ORF103	1.002	C1ORF38	0.757		
ITGB7	1.513	NPC2	0.996	FAM122C	0.752		

293T cells were transfected with plasmids producing VSV-G pseudotyped NL4-3luc, together with an empty vector or a plasmid expressing an ISG. A plasmid expressing renilla luciferase was included to serve as a control for transfection efficiency and sample handling. At 48 h posttransfection, the producer cells were analyzed for luciferase activities and the culture supernatants were used to infect recipient cells. At 48 h postinfection, firefly luciferase activity in the recipient cells was measured and normalized by the renilla luciferase activity in the producer cells. Fold inhibition was calculated as normalized luciferase activity in recipient cells infected with the virus produced from the control cells divided by that from ISG-expressing cells.
